# "Flora of Russia" on iNaturalist: a dataset

**DOI:** 10.3897/BDJ.8.e59249

**Published:** 2020-11-17

**Authors:** Alexey P. Seregin, Dmitriy A. Bochkov, Julia V. Shner, Eduard V. Garin, Igor N. Pospelov, Vadim E. Prokhorov, Pavel V. Golyakov, Sergey R. Mayorov, Sergey A. Svirin, Alexander N. Khimin, Marina S. Gorbunova, Ekaterina S. Kashirina, Olga P. Kuryakova, Boris V. Bolshakov, Aleksandr L. Ebel, Anatoliy A. Khapugin, Maxim M. Mallaliev, Sergey V. Mirvoda, Sergey A. Lednev, Dina V. Nesterkova, Nadezhda P. Zelenova, Svetlana A. Nesterova, Viktoriya N. Zelenkova, Georgy M. Vinogradov, Olga V. Biryukova, Alla V. Verkhozina, Alexey P. Zyrianov, Sergey V. Gerasimov, Ramazan A. Murtazaliev, Yurii M. Basov, Kira Yu. Marchenkova, Dmitry R. Vladimirov, Dina B. Safina, Sergey V. Dudov, Nikolai I. Degtyarev, Diana V. Tretyakova, Daba G. Chimitov, Evgenij A. Sklyar, Alesya N. Kandaurova, Svetlana A. Bogdanovich, Alexander V. Dubynin, Olga A. Chernyagina, Aleksandr V. Lebedev, Mikhail S. Knyazev, Irina Yu. Mitjushina, Nina V. Filippova, Kseniia V. Dudova, Igor V. Kuzmin, Tatyana Yu. Svetasheva, Vladimir P. Zakharov, Vladimir P. Travkin, Yaroslav O. Magazov, Vladimir Yu. Teploukhov, Andrey N. Efremov, Olesya V. Deineko, Viktor V. Stepanov, Eugene S. Popov, Dmitry V. Kuzmenckin, Tatiana L. Strus, Tatyana V. Zarubo, Konstantin V. Romanov, Alexei L. Ebel, Denis V. Tishin, Vladimir Yu. Arkhipov, Vladimir N. Korotkov, Svetlana B. Kutueva, Vladimir V. Gostev, Mikhail M. Krivosheev, Natalia S. Gamova, Veronica A. Belova, Oleg E. Kosterin, Sergey V. Prokopenko, Rinat R. Sultanov, Irina A. Kobuzeva, Nikolay V. Dorofeev, Alexander A. Yakovlev, Yuriy V. Danilevsky, Irina B. Zolotukhina, Damir A. Yumagulov, Valerii A. Glazunov, Vladimir A. Bakutov, Andrey V. Danilin, Igor V. Pavlov, Elena S. Pushay, Elena V. Tikhonova, Konstantin V. Samodurov, Dmitrii V. Epikhin, Tatyana B. Silaeva, Andrei I. Pyak, Yulia A. Fedorova, Evgeniy S. Samarin, Denis S. Shilov, Valentina P. Borodulina, Ekaterina V. Kropocheva, Gennadiy L. Kosenkov, Uladzimir V. Bury, Anna E. Mitroshenkova, Tatiana A. Karpenko, Ruslan M. Osmanov, Maria V. Kozlova, Tatiana M. Gavrilova, Stepan A. Senator, Maxim I. Khomutovskiy, Eugene A. Borovichev, Ilya V. Filippov, Serguei V. Ponomarenko, Elena A. Shumikhina, Dmitry F. Lyskov, Evgeny A. Belyakov, Mikhail N. Kozhin, Leonid S. Poryadin, Artem V. Leostrin

**Affiliations:** 1 M.V. Lomonosov Moscow State University, Moscow, Russia M.V. Lomonosov Moscow State University Moscow Russia; 2 Lomonosov Moscow State University, Moscow, Russia Lomonosov Moscow State University Moscow Russia; 3 Papanin Institute for Biology of Inland Waters, RAS, Borok, Yaroslavl Oblast, Russia Papanin Institute for Biology of Inland Waters, RAS Borok, Yaroslavl Oblast Russia; 4 Severtsov Institute of Ecology and Evolution, RAS, Moscow, Russia Severtsov Institute of Ecology and Evolution, RAS Moscow Russia; 5 Kazan Federal University, Kazan, Russia Kazan Federal University Kazan Russia; 6 Tigirek State Reserve, Barnaul, Russia Tigirek State Reserve Barnaul Russia; 7 Sevastopol State University, Sevastopol, Russia Sevastopol State University Sevastopol Russia; 8 Pavlovsk Secondary School #2, Pavlovsk, Voronezh Oblast, Russia Pavlovsk Secondary School #2 Pavlovsk, Voronezh Oblast Russia; 9 Independent Researcher, Korolyov, Moscow Oblast, Russia Independent Researcher Korolyov, Moscow Oblast Russia; 10 Lomonosov Moscow State University (Sevastopol Branch), Sevastopol, Russia Lomonosov Moscow State University (Sevastopol Branch) Sevastopol Russia; 11 Independent Researcher, Milkovo, Kamchatsky Krai, Russia Independent Researcher Milkovo, Kamchatsky Krai Russia; 12 Independent Researcher, Petropavlovsk-Kamchatsky, Russia Independent Researcher Petropavlovsk-Kamchatsky Russia; 13 Tomsk State University, Tomsk, Russia Tomsk State University Tomsk Russia; 14 Joint Directorate of the Mordovia State Nature Reserve and National Park “Smolny”, Saransk, Russia Joint Directorate of the Mordovia State Nature Reserve and National Park “Smolny” Saransk Russia; 15 Tyumen State University, Tyumen, Russia Tyumen State University Tyumen Russia; 16 Mountain Botanical Garden, Dagestan Federal Research Centre, RAS, Makhachkala, Russia Mountain Botanical Garden, Dagestan Federal Research Centre, RAS Makhachkala Russia; 17 Belorechenskoye Agricultural Public Joint Stock Company, Belorechensky, Irkutsk Oblast, Russia Belorechenskoye Agricultural Public Joint Stock Company Belorechensky, Irkutsk Oblast Russia; 18 Institute of Plant and Animal Ecology, Ural Branch, RAS, Ekaterinburg, Russia Institute of Plant and Animal Ecology, Ural Branch, RAS Ekaterinburg Russia; 19 Independent Researcher, Kaliningrad, Russia Independent Researcher Kaliningrad Russia; 20 Independent Researcher, Kostroma, Russia Independent Researcher Kostroma Russia; 21 Belgorod National Research University, Belgorod, Russia Belgorod National Research University Belgorod Russia; 22 Shirshov Institute of Oceanology, RAS, Moscow, Russia Shirshov Institute of Oceanology, RAS Moscow Russia; 23 Lobachevsky State University, Nizhny Novgorod, Russia Lobachevsky State University Nizhny Novgorod Russia; 24 Siberian Institute of Plant Physiology and Biochemistry, SB RAS, Irkutsk, Russia Siberian Institute of Plant Physiology and Biochemistry, SB RAS Irkutsk Russia; 25 Independent Researcher, Novosibirsk, Russia Independent Researcher Novosibirsk Russia; 26 Republican Children’s Ecological and Biological Center of Bashkortostan, Ufa, Russia Republican Children’s Ecological and Biological Center of Bashkortostan Ufa Russia; 27 Dagestan State Medical University, Makhachkala, Russia Dagestan State Medical University Makhachkala Russia; 28 Independent Researcher, Tyumen, Russia Independent Researcher Tyumen Russia; 29 Independent Researcher, Bryansk, Russia Independent Researcher Bryansk Russia; 30 Voronezh State University, Voronezh, Russia Voronezh State University Voronezh Russia; 31 Independent Researcher, Sevastopol, Russia Independent Researcher Sevastopol Russia; 32 Central Chernozem Reserve, Kursk Oblast, Russia Central Chernozem Reserve Kursk Oblast Russia; 33 Togliatti State University, Togliatti, Russia Togliatti State University Togliatti Russia; 34 Institute of General and Experimental Biology, RAS, Ulan-Ude, Russia Institute of General and Experimental Biology, RAS Ulan-Ude Russia; 35 Independent Researcher, Kursk, Russia Independent Researcher Kursk Russia; 36 Independent Researcher, Saratov, Russia Independent Researcher Saratov Russia; 37 Independent Researcher, Alushta, Russia Independent Researcher Alushta Russia; 38 Open Laboratory of Conservation Biology, Novosibirsk, Russia Open Laboratory of Conservation Biology Novosibirsk Russia; 39 Novosibirsk State University, Novosibirsk, Russia Novosibirsk State University Novosibirsk Russia; 40 Kamchatka Branch of the Pacific Geographical Institute, FEB RAS, Petropavlovsk-Kamchatsky, Russia Kamchatka Branch of the Pacific Geographical Institute, FEB RAS Petropavlovsk-Kamchatsky Russia; 41 Vitus Bering Kamchatka State University, Petropavlovsk-Kamchatsky, Russia Vitus Bering Kamchatka State University Petropavlovsk-Kamchatsky Russia; 42 Russian State Agrarian University - Moscow Timiryazev Agricultural Academy, Moscow, Russia Russian State Agrarian University - Moscow Timiryazev Agricultural Academy Moscow Russia; 43 Institute Botanic Garden, Ural Branch, RAS, Ekaterinburg, Russia Institute Botanic Garden, Ural Branch, RAS Ekaterinburg Russia; 44 Directorate of Protected Areas, Vladimir, Russia Directorate of Protected Areas Vladimir Russia; 45 Yugra State University, Khanty-Mansiysk, Russia Yugra State University Khanty-Mansiysk Russia; 46 Tula State Lev Tolstoy Pedagogical University, Tula, Russia Tula State Lev Tolstoy Pedagogical University Tula Russia; 47 Independent Researcher, Likino-Dulyovo, Moscow Oblast, Russia Independent Researcher Likino-Dulyovo, Moscow Oblast Russia; 48 Independent Researcher, Samara, Russia Independent Researcher Samara Russia; 49 Chelyabinsk Children and Youth Camping Trip Centre "Kosmos", Chelyabinsk, Russia Chelyabinsk Children and Youth Camping Trip Centre "Kosmos" Chelyabinsk Russia; 50 Independent Researcher, Omsk, Russia Independent Researcher Omsk Russia; 51 Ulyanovsk State Pedagogical University, Ulyanovsk, Russia Ulyanovsk State Pedagogical University Ulyanovsk Russia; 52 Independent Researcher, Vladimir, Russia Independent Researcher Vladimir Russia; 53 Komarov Botanical Institute, RAS, Saint Petersburg, Russia Komarov Botanical Institute, RAS Saint Petersburg Russia; 54 Independent Researcher, Khanty-Mansiysk, Russia Independent Researcher Khanty-Mansiysk Russia; 55 Independent Researcher, Nizhny Novgorod, Russia Independent Researcher Nizhny Novgorod Russia; 56 Altai State University, Barnaul, Russia Altai State University Barnaul Russia; 57 Institute of Theoretical and Experimental Biophysics, RAS, Pushchino, Russia Institute of Theoretical and Experimental Biophysics, RAS Pushchino Russia; 58 Izrael Institute of Global Climate and Ecology, Moscow, Russia Izrael Institute of Global Climate and Ecology Moscow Russia; 59 Rybinsk State Historical, Architectural and Art Museum Preserve, Rybinsk, Yaroslavl Oblast, Russia Rybinsk State Historical, Architectural and Art Museum Preserve Rybinsk, Yaroslavl Oblast Russia; 60 Bashkir State University, Ufa, Russia Bashkir State University Ufa Russia; 61 Baikalsky State Nature Biosphere Reserve, Tankhoy, Buryat Republic, Russia Baikalsky State Nature Biosphere Reserve Tankhoy, Buryat Republic Russia; 62 Irkutsk State University, Irkutsk, Russia Irkutsk State University Irkutsk Russia; 63 Institute of Cytology and Genetics, SB RAS, Novosibirsk, Russia Institute of Cytology and Genetics, SB RAS Novosibirsk Russia; 64 Federal Scientific Center of the East Asia Terrestrial Biodiversity, FEB RAS, Vladivostok, Russia Federal Scientific Center of the East Asia Terrestrial Biodiversity, FEB RAS Vladivostok Russia; 65 Independent Researcher, Moscow, Russia Independent Researcher Moscow Russia; 66 Independent Researcher, Cheboksary, Russia Independent Researcher Cheboksary Russia; 67 Independent Researcher, Ust-Labinsk, Krasnodar Krai, Russia Independent Researcher Ust-Labinsk, Krasnodar Krai Russia; 68 Independent Researcher, Ufa, Russia Independent Researcher Ufa Russia; 69 Institute of the Problems of Northern Development, Tyumen Scientific Centre SB RAS, Tyumen, Russia Institute of the Problems of Northern Development, Tyumen Scientific Centre SB RAS Tyumen Russia; 70 Independent Researcher, Volzhsk, Mari El Republic, Russia Independent Researcher Volzhsk, Mari El Republic Russia; 71 NUST MISIS, Moscow, Russia NUST MISIS Moscow Russia; 72 Independent Researcher, Perm Krai, Russia Independent Researcher Perm Krai Russia; 73 Tver State University, Tver, Russia Tver State University Tver Russia; 74 Center for Forest Ecology and Productivity, RAS, Moscow, Russia Center for Forest Ecology and Productivity, RAS Moscow Russia; 75 Independent Researcher, Asino, Tomsk Oblast, Russia Independent Researcher Asino, Tomsk Oblast Russia; 76 Vernadsky Crimean Federal University, Simferopol, Russia Vernadsky Crimean Federal University Simferopol Russia; 77 Ogarev Mordovia State University, Saransk, Russia Ogarev Mordovia State University Saransk Russia; 78 Tuva State University, Kyzyl, Russia Tuva State University Kyzyl Russia; 79 Institute of Biology, Ufa Federal Research Centre, RAS, Ufa, Russia Institute of Biology, Ufa Federal Research Centre, RAS Ufa Russia; 80 Independent Researcher, Neftekamsk, Russia Independent Researcher Neftekamsk Russia; 81 Visim State Natural Biosphere Reserve, Pervouralsk, Sverdlovsk Oblast, Russia Visim State Natural Biosphere Reserve Pervouralsk, Sverdlovsk Oblast Russia; 82 Institute of Molecular Genetics, National Research Centre «Kurchatov Institute», Moscow, Russia Institute of Molecular Genetics, National Research Centre «Kurchatov Institute» Moscow Russia; 83 Sebezhsky Museum of Local Lore, Sebezh, Pskov Oblast, Russia Sebezhsky Museum of Local Lore Sebezh, Pskov Oblast Russia; 84 Samara State University of Social Sciences and Education, Samara, Russia Samara State University of Social Sciences and Education Samara Russia; 85 Zubov State Oceanographic Institute, Moscow, Russia Zubov State Oceanographic Institute Moscow Russia; 86 Tsitsin Main Botanical Garden, RAS, Moscow, Russia Tsitsin Main Botanical Garden, RAS Moscow Russia; 87 Institute of Ecology of the Volga River Basin, Samara Federal Research Center, RAS, Togliatti, Russia Institute of Ecology of the Volga River Basin, Samara Federal Research Center, RAS Togliatti Russia; 88 Institute of North Industrial Ecology Problems, Kola Science Centre, RAS, Apatity, Russia Institute of North Industrial Ecology Problems, Kola Science Centre, RAS Apatity Russia; 89 Canadian Museum of Nature, Ottawa, Canada Canadian Museum of Nature Ottawa Canada; 90 Avrorin Polar-Alpine Botanical Garden-Institute, RAS, Apatity, Russia Avrorin Polar-Alpine Botanical Garden-Institute, RAS Apatity Russia

## Abstract

**Background:**

The "Flora of Russia" project on iNaturalist brought together professional scientists and amateur naturalists from all over the country. Over 10,000 people were involved in the data collection.

**New information:**

Within 20 months, the participants accumulated 750,143 photo observations of 6,857 species of the Russian flora. This constitutes the largest dataset of open spatial data on the country’s biodiversity and a leading source of data on the current state of the national flora. About 87% of all project data, i.e. 652,285 observations, are available under free licences (CC0, CC-BY, CC-BY-NC) and can be freely used in scientific, educational and environmental activities.

## Introduction

Since 2008, iNaturalist (https://www.inaturalist.org/) has been crowdsourcing biodiversity observations made by citizen scientists, as well as their taxonomic identifications. Hundreds of publications have already taken into account iNaturalist data for use in research, conservation and policy (e.g. Ocampo-Peñuela et al. 2016, Chandler et al. 2017, Heberling and Isaac 2018). There are three key themes that iNaturalist embraces: social interaction; shareability of data, tools and code; and scalability of the platform and community (Seltzer 2019).

The advent of large, technology-based resources allows ecologists and biologists to work at spatio-temporal scales previously unimaginable (White et al. 2015). With 50M observations accompanied by photo or audio evidence, the global iNaturalist dataset is one of the largest online collections of biodiversity data. It is partially represented in the GBIF, with the exclusion of observations which remain unidentified or have unconfirmed or missing licence information. Nonetheless, the GBIF export tools provide excellent data usability and the resulting exports come with a DOI which one can use for citation in publications. The GBIF data usage counter shows that iNaturalist GBIF-mediated data gained 781 citations (as of 11 Sep 2020) making it one of the most commonly-used datasets amongst the GBIF (Ueda 2020).

Many research papers focus on the employment of iNaturalist data as a primary source (Heberling and Isaac 2018, Seregin et al. 2020). For instance, iNaturalist includes dozens of metadata fields for every observation and was employed as a case study in the theory of long-tailed datasets (Cui et al. 2019). Observations from the iNaturalist Challenge at FGVC 2017 with links to 675,000 licensed images of 5,089 species have been widely used in computer vision training (Cui et al. 2018; Van Horn et al. 2018; Zheng et al. 2019). iNaturalist observations and images have been be employed as a data source in classical taxonomy of tracheophytes (Svoboda and Harris 2018), studies of the distribution of gecko clones (Lapwong and Juthong 2018), plant phenology (Barve et al. 2020) and fish infections on a continental scale (Happel 2019). Moreover, Skejo et al. (2020) recently published a description of a new species, based on photos from iNaturalist in addition to scarce museum material. The platform has been suggested as a suitable agent for storage of photo vouchers associated with museum specimens (Heberling and Isaac 2018).

Biodiversity documentation, by the means of aggregation of individual observations, is the main goal of iNaturalist. Consistent with this are the many examples of papers dealing with new noteworthy records of either alien (Vendetti et al. 2018; Hiller and Haelewaters 2019; Liebgold 2019) or native organisms (Rosenberg 2018; Schuette et al. 2018) made by amateur naturalists. Further accumulation of data made possible precise documentation of alien species distribution on a nationwide scale (Ciceoi et al. 2017), their expansion process (Oficialdegui et al. 2020), routine monitoring of invasive species (Larson et al. 2020), documentation of at-risk species beyond the boundaries of protected areas (Young et al. 2019) and a global assessment of species’ extinction risk with the inclusion of citizen science data (Gardiner and Bachman 2016). Spatial data from iNaturalist have been employed in studies of bird collisions with windows (Winton et al. 2018), global snakebite mortality (Longbottom et al. 2018) and the search for environmental triggers in orchids (Lori et al. 2018).

It has recently been shown that iNaturalist serves as a tool indispensable for avoiding biases in urban biodiversity data (Li et al. 2019), for making decisions related to the urban management of red foxes and coyotes (Mueller et al. 2019) and for testing urban biotic homogenisation with the use of data generated by the participants of the City Nature Challenge (Leong and Trautwein 2019). There are positive examples of iNaturalist usage in data accumulation by researchers (Ocampo-Peñuela et al. 2016), as well as the citizen community helping scientists with a supply of data (Brown et al. 2019). In addition, there are examples of iNaturalist usage during university courses of classical zoology and botany together with standard field guides and keys (Unger et al. 2020).

The iNaturalist dataset at various taxonomic and/or geographical extents has been checked for completeness of data against complete literature data (Goldstein et al. 2018), expert-based range maps (Fourcade 2016), museum collections (Spear et al. 2017) and available inventories within protected areas (Jacobs and Zipf 2017). Vahidi et al. (2017) performed a general quality assessment of iNaturalist data which made possible the revealing of the majority of attribute and positional errors amongst the crowd-sourced biodiversity observations. Borzée et al. (2019) published a case study on cross-verification of iNaturalist observations against published georeferenced molecular data, whereas Maritz and Maritz (2020) compared Facebook versus iNaturalist as data sources in the assessment of trophic interactions. Prudic et al. (2018) verified the completeness of iNaturalist data with various field techniques of butterfly data collection.

**Figure 1.**
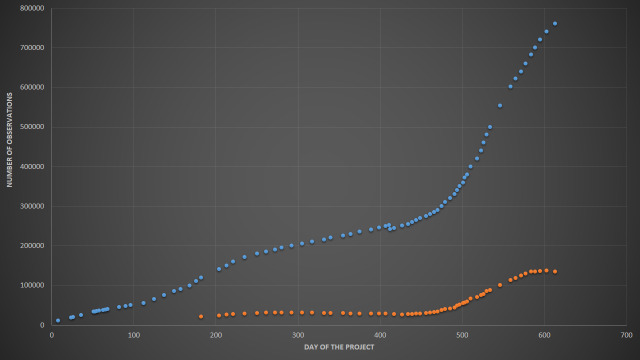
Dynamics of identified and verified ("research-grade") observations of the "Flora of Russia" project since the inception. Blue dots represent the research grade observations and red dots correspond to unverified observations from the project's backlog. About 11K observations were deleted from iNaturalist by a single "mega-observer" on 25 Feb 2020.

**Figure 2.**
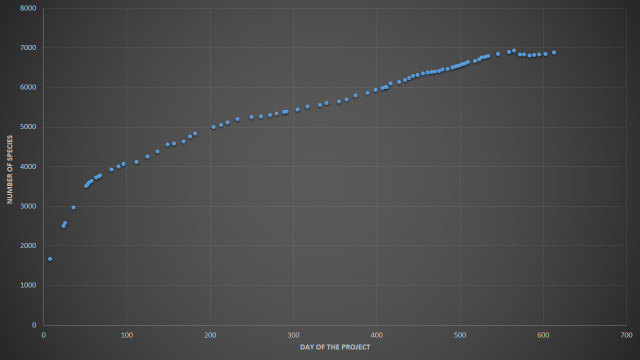
The "Flora of Russia" project species number dynamics since the inception on 9 Jan 2019. From 31 Jul 2020, the number of species stabilised due to ongoing expert data cleaning activity.

**Figure 3.**
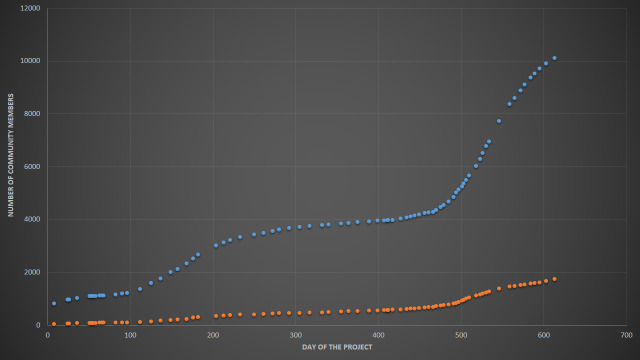
Observers (blue dots) and members (red dots) of the "Flora of Russia" project since the inception on 9 Jan 2019.

The project "Flora of Russia", which includes all verified ("research-grade") observations of vascular plants from the country, was launched by the Moscow University team on 9 Jan 2019 to support data collection for the "Atlas of the Russian flora" (Seregin et al. 2020). During the first 20 months, the number of identified and verified iNaturalist observations of vascular plants from Russia increased 68-fold and the number of involved users increased 10-fold. Here, we present the characteristics of the dataset as for 9 Sep 2020, soon after the project reached two notable milestones of 750,000 verified observations and 10,000 observers (Fig. 1, Fig. 2 and Fig. 3).

Fig. 3 shows both the number of observers and project members. Since the collection projects on iNaturalist are working as filters, all RG observations of vascular plants from Russia are covered by the project giving an impressive figure of 10K observers. As of 13 Sep 2020, 1,736 members of iNaturalist have formally joined the "Flora of Russia" project by pressing the "Join" button. As a result, they clearly affiliate their data with the project by an automatically-generated logo on every observation page and receive notifications on project updates and journal posts. Those observers who are not members of the project still get benefits in the form of identifications, because experts are inspecting all observations available on iNaturalist.

## General description

### Purpose

For a number of years, Russian professional and amateur biologists were using Internet-based national networking systems of the georeferenced data collection for birds, invertebrates and plants. For instance, Plantarium is the most popular Russian-language resource for collecting plant and lichen photographs from around the world with emphasis on Russia and adjacent regions. However, unlike iNaturalist, it does not allow data export nor is these data included in the GBIF, since photos and other data lack licence indications. In addition, contributing observations to Plantarium requires more effort from the members.

After digitisation of the nation's second largest herbarium (Seregin 2018), the Moscow University team launched a public awareness campaign to support community-generated data collection for plants. We decided not to spend budget on our own crowd-sourcing system, but to use and promote the international iNaturalist platform as suitable for data collection in Russia with a number of efficient tools and a global community.

**Russia on iNaturalist**: By late 2018, Russia was the 18th country on iNaturalist in terms of the number of verifiable observations (47,888). After 20 months of the project activity, we can see drastic changes in the biodiversity data coverage across Russia with a strong emphasis on tracheophytes.

Currently, Russia holds fifth place amongst countries represented on iNaturalist in terms of the number of verifiable observations of all groups of organisms and the third place by observations of vascular plants in particular (Table 1).

Amongst the top ten countries, Russia has the highest proportion of tracheophyte observations of all uploaded to iNaturalist (62.2%). A community of birdwatchers is also quite active when compared to other top countries, whereas other groups of organisms are still lacking much attention (Table 2). Birds are the primary object of attention for at least eight co-authors of this paper, whereas three of us are focused on fungi.

Russia has the highest proportion of vascular plants amongst identified and confirmed observations which are classified as "research grade" on iNaturalist (Table 3). Moreover, Russia is the leading country on iNaturalist amongst the top ten with regard to the proportion of confirmed observations amongst all tracheophyte records. As we showed in 2019 (Seregin et al. 2020), the number of unconfirmed plant observations in Russia usually rapidly increases from May to August and decreases from September to April, when experts most intensively work with the backlog of unprocessed observations.

To facilitate further accumulation of the project's data into the GBIF, we ask our observers to specify open Creative Commons Licences, such as CC0 (http://creativecommons.org/publicdomain/zero/1.0/), 2) CC-BY (http://creativecommons.org/licenses/by/4.0/) and 3) CC-BY-NC (http://creativecommons.org/licenses/by-nc/4.0/) for their observations. We do this on a regular basis in the form of the project's journal posts available to every member of our community. As a result of this activity, 83.8% of all observations on iNaturalist from Russia (and as many as 85.3% in tracheophytes) are freely licensed, making Russia the leader in open-access biodiversity data on iNaturalist (Table 4).

As a result of intense expert activity and the promotion of free licensing, 73.8% of tracheophyte records from Russia have become available in the GBIF (Table 5) which is the highest proportion amongst the leading countries in iNaturalist. Since 2020, the iNaturalist dataset has become the largest source of data on the Russian biodiversity available through the GBIF.

The number of observers with at least a single verifiable observation is not so high in Russia, equalling just 14K (Table 6). Nonetheless, the average productivity of the members of the community is extremely high. On average, 93 verifiable observations have been created by each observer across all groups of organisms, while, with regard to vascular plants, the number is 73 "research-grade" observations per observer, which makes the highest level of observer activity amongst the top ten countries on iNaturalist.

Russia is globally unique taking into account the active growth of data within the "Flora of Russia" project. Amongst the top ten countries on iNaturalist, Russia has achieved:

1. the highest proportion of tracheophytes amongst all observations;

2. the highest proportion of identified and verified ("research-grade") observations amongst tracheophytes;

3. the highest proportion of both free licences (CC0, CC-BY & CC-BY-NC) and GBIF records;

4. the highest number of observations per observer.

## Project description

### Title

"Flora of Russia" project on iNaturalist

### Personnel

As of 13 Sep 2020, 1,736 members of iNaturalist have joined the project (see also Fig. 3). The core of the project team is formed by 129 people, who are listed simultaneously amongst the top 200 identifiers and top 500 observers of the project, including 15 project members affiliated with the Lomonosov Moscow State University. Of the 129 members, 112 confirmed their formal contribution to this data paper (see the "Author contributions" section and the "Community coverage" section for additional information). Dr. Alexey P. Seregin is the founder and an administrator of the project.

### Study area description

The project covers the territory of the Russian Federation as defined by the national legislation, i.e. including the Republic of Crimea and the City of Sevastopol claimed by Ukraine.

### Design description

**Main features of iNaturalist as a data collection platform**

Any user can register as an "observer" on iNaturalist. Users may upload observations of organisms through their account using the website https://www.inaturalist.org/ or the free mobile applications "iNaturalist" and "Seek". A total of 1.28M observers are involved in the work of the platform, including 14.3K observers with at least one observation from Russia (Table 6).

In order to meet the minimum requirements for further scientific use, an observation needs to have: (1) a date; (2) a georeference; (3) a photograph/series of photographs or (for animals) an audio recording(s) of the object's sounds, created by the observer; (4) the organism needs to be recorded in the wild. Provided that these requirements are fulfilled, the observation is marked as "needs ID", regardless of whether the author identified the organism or not. Once an observation receives identical identifications by more than two thirds of the iNaturalist users at the level of species (in some cases of genus), it becomes "research-grade", a category for verified observations. A supporting identification by a second user makes an observation "research-grade" while identification by a single user is not enough. Disagreeing identifications may once again exclude an observation from this category. Low-quality photos or photos of plants accurate identification of which requires a study of some micromorphological, anatomical or genetic traits usually do not reach the "research grade" or, in the latter cases, remain identified and verified only at the level of genus.

The observers may choose a licence allowing further re-use of the data. Observations licensed with three Creative Commons Licences (CC0, CC-BY, CC-BY-NC) and of "research-grade" quality are automatically exported to the GBIF. As of 5 Sep 2020, the iNaturalist database contained 48.6M observations that have met the minimum quality requirements (Table 1), of which 29.2M have achieved "research-grade" (Table 3). Unfortunately, only 19.7M observations have been exported to the GBIF due to copyright restrictions (Table 5).

The implementation of artificial intelligence (AI) for identification is a key feature of iNaturalist (Van Horn et al. 2018, Cui et al. 2018). It gives the users a suggestion about the most similar species after analysing the photos ("visually similar") and taking into account the geographical distribution of other records ("seen nearby"). Initially, the portal's AI compared the newly-uploaded photos with a basic set of images, which, in 2017, comprised 859,000 photos of more than 5,000 species. The images of varying quality had been collected using different types of cameras, but their identifications have been double-checked. Primary results showed that modern AI methods, at that time, gave an accurate identification for 67% of observations, which well illustrates the complexity of the dataset (Van Horn et al. 2018). In 2018, most images of plants and animals from any part of the world were likely to receive from the system an identification of species inhabiting North America. Over the course of 2019 and 2020, AI has almost stopped suggesting incorrect identifications for plants within European Russia (Seregin et al. 2020). It still works somewhat worse with photos from Asian Russia and the Caucasus. Millions of new photos reviewed by the expert community and constantly added to the library of standard images allow AI to improve the performance. Its capabilities are, however, still inferior to expert assessments with regard to certain groups of organisms or certain geographic territories. Nevertheless, the system's general awareness of the world flora is many times larger than that of an individual botanist. In many ways, this particular feature of iNaturalist attracts both amateurs and professionals. The success of iNaturalist has made possible the further use of AI for species recognition by photograph for millions of images in the GBIF database (Robertson et al. 2019).

**Portal on the flora of Russia**

To collect data on the plant distribution in the City of Moscow, the Moscow University team initially organised the "Flora of Moscow" project on iNaturalist on 29 Dec 2018. An immediate positive feedback from users and a surge of interest forced us to create 85 more regional projects with a uniform ideology in early January 2019 and organise them as part of the "Flora of Russia" umbrella project. Each regional portal automatically includes observations of vascular plants uploaded on iNaturalist which have achieved "research-grade" and found within the administrative boundaries of a specific federal territory. The home page of each regional portal displays its statistics and basic information.

**Figure 4.**
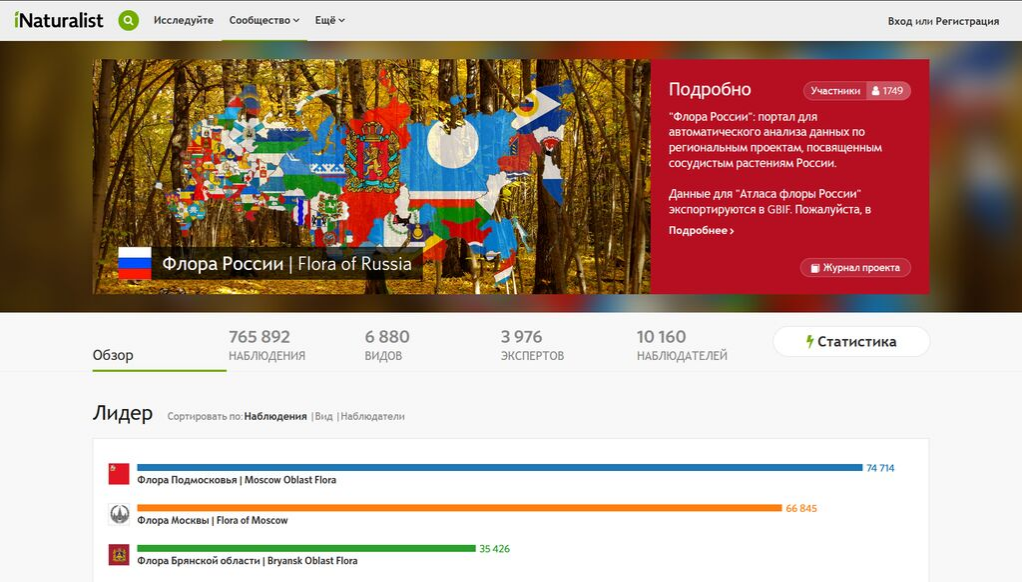
The homepage of the "Flora of Russia" project on iNaturalist (Russian-language interface, statistics as of 15 Sep 2020).

The "Flora of Russia" homepage (Fig. 4) includes a "scoreboard" with a ranking of regional projects (ordered by the number of observations, species and observers), basic statistics, a list of the latest observations, news from the project journal and a general map of all data. There are links leading to the project description, the project journal, the rankings of the top observers (ordered by the number of observations and species), top identifiers, most often recorded species and detailed statistical reports. Thus, both regional projects and the all-Russian portal are organised in the form of ranking tables, stimulating both individual and team activity of observers in accordance with the gamification paradigm (Bowser et al. 2013).

The experts (most of whom are the authors of this paper) review the unverified and unnamed observations to suggest the correct name which may either confirm or disprove the opinion of the observer. Typically, most clear photographs from European Russia and the Russian Far East are identified within a couple days after uploading.

### Funding

The project is functioning on a voluntary basis. Although being created in the Lomonosov Moscow State University, it does not have formal institutional funding. Members of the project search for their own budget for field trips and online activity. Some grants of the co-authors are acknowledged in this paper.

## Sampling methods

### Sampling description

The standard procedure of sampling is described on iNaturalist in the form of 17 paragraphs in the "Observations" section of the help page (last revised 8 Sep 2020 by Sam Kieschnick).

### Quality control

Data quality control is necessary for maintaining a high quality of records within a dataset. In the "Flora of Russia" project description, there is a well-structured, detailed and constantly improving section with recommendations for users in Russian. Apart from the general information (including short videos about iNaturalist and a description of available research tools of the portal), there are two particularly important sections, i.e. "Recommendations for new users" and "Recommendations for event curators". Both sections provide detailed instructions for the user on what, how and where to create a good-quality observation on iNaturalist. However, many users are not familiar with these guidelines. This imposes a certain responsibility on the identifiers and the project curators, who act as data stewards. The most important and/or frequently occurring issues are listed below.

For each project on iNauralist, at least one or two curators should be assigned to review the uploaded observations and make comments, if necessary. The most frequent mistakes are:

1. low-quality or wrong-angle photos,
2. observations of cultivated plants without a relevant indication,
3. either unintentional or intentional duplication of the same observation,
4. unintentional merging of numerous observations into a single one,
5. lack of date or location of an observation,
6. lack of any original identification (at least a coarse one),
7. upload of copyright media.

In some cases, an inaccurate location for an observation shows up automatically, caused by specific GPS settings on the smartphone or camera. We report these issues to observers for further manual correction or mark such observations as "location is not accurate". We highly recommend georeferencing using the "GPS only" mode instead of either "GPS plus mobile networks" and "mobile networks only". The latter two options may shift the observation's georeference to the nearest mobile tower instead of the actual observer's location. Additionally, all records with positional accuracy exceeding 50,000 m were marked as having inaccurate location on 25 Sep 2020 and reported to users in the project journal post. Suspicious positional accuracy of 0, 1 or 2 metres recorded in thousands of observations is an artifact set up automatically during the uploading of observations by the devices.

Another difficult and common problem is the separation of cultivated plants from garden escapes (naturalised or casual). Cultivated plants may be well recognisable and could reach "research grade" rapidly. We ask experts and project curators to double-check "research-grade" observations to detect plants growing only in cultivation.

A well-designed and useful feature in iNaturalist is the possibility to call for attention of a specific user using the "@" prefix (for example, @krestov). This is very important for maintaining the appropriate quality as experts may respond and help in identification.

Undoubtedly, the data quality depends on the quality of the uploaded photographs and field experience of the users. We ask project curators to post links to regional checklists, field guides and illustrated atlases for interested naturalists in the project description.

Constant quality control is especially important during various events such as bioblitzes or mandatory student practices. As their numerous participants mostly lack experience in collecting biodiversity data through iNaturalist, the work of curators and teachers should be constant during the whole period of these events.

## Geographic coverage

### Description

**Figure 5.**
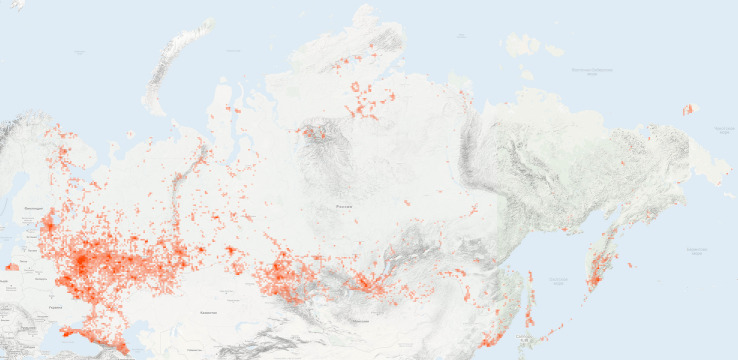
A map of 750K observations from the "Flora of Russia" project showing an extreme disproportion in data coverage (source: iNaturalist.org).

Russia is a large country with an area of over 17 million km^2^ and an unevenly distributed human population. For instance, in Chukotka, the population density is only 0.07 people per 1 km^2^, whereas in the City of Moscow, it is 4,925 people per 1 km^2^ (Table 7). The geographic coverage of the dataset is characterised by significant spatial disparities in the presented data for all indexes, including the number of observations, species and observers (Fig. 5).

**Number of observations.** The key index of the "Flora of Russia" project is the number of uploaded observations (Fig. 1). The project reached **750,000 observations** of "research-grade" quality on 7 Sep 2020, whereas ca. 135,000 unverified observations make the project's backlog, which is not included in the dataset. The stable snapshot of the dataset produced on 8 Sep 2020 contains 750,143 records (see "Data resources" section).

The City of Moscow topped the project by the number of observations from 18 Aug 2019 to 15 Jun 2020, when Moscow Oblast, the region with the largest community of observers, took the lead (Table 8). Other regions of Central Russia - Bryansk, Tula, Nizhny Novgorod and Kursk Oblasts - hold the third to sixth places in the ranking.

The top 10 regional projects contribute 45.4% of observations of the entire project and this proportion is constantly decreasing due to the growth of the communities in other regions. For instance, the proportion of observations made in the top ten regions was 55.5% on 9 Jan 2020. However, the disproportion in the spatial coverage is obvious even within the leading regions (Fig. 6).

Less than 500 observations have been made within each of the four regions of the Caucasian biodiversity hotspot (Kabardino-Balkaria, Chechen Republic, North Ossetia, Ingushetia), desert regions (Astrakhan Oblast, Kalmykia), as well as in Magadan Oblast, Tyva Republic, Jewish Autonomous Oblast and Nenets Autonomous Okrug.

**Observations per capita.** If we normalise the number of observations per 1,000 inhabitants, it turns out that the two most active communities are in the City of Sevastopol and Kamchatka, followed by Bryansk Oblast, Kursk Oblast, Mordovia, Kostroma Oblast and, unexpectedly, Chukotka, a vast region with a very small population. In general, this index best reflects both the involvement of the local residents in the "Flora of Russia" project and the activity of this particular region's community.

**Observation density.** Spatial sampling is best characterised by the density of observations per a standard area (for example, per 1,000 km^2^). The three federal cities are far ahead: Moscow (26K observations), Sevastopol (25K) and St. Petersburg (7K), here, a large urban population is concentrated on a small area. Federal cities are followed by four regions of Central Russia with a relatively small area and active local iNaturalist communities, i.e. Moscow Oblast, Tula Oblast, Chuvashia and Bryansk Oblast.

**Observations per recorded species.** The number of observations per recorded species is the integrated index which best characterises both the data density and species representation. The gradual accumulation of observations leads to consequent revealing of all known species or, at least, of regularly observed plants. When recording a new species becomes a rare event and an active community still posts many new photos, the average number of observations per species begins to grow rapidly. According to this index, the leaders are Moscow Oblast (65), City of Moscow (61), Bryansk Oblast (31), Tula Oblast (29), Nizhny Novgorod Oblast (27) and Omsk Oblast (25). Regions with rich floras (for example, montainous areas) outperform the relatively-poor plains because more observations need to be made there to record numerous rare species.

### Coordinates

41 and 82 Latitude; 19.5 and -169 Longitude.

## Taxonomic coverage

### Description

As of 7 Sep 2020, the "Flora of Russia" project included observations of **6,857 species** of vascular plants (Fig. 2). Plants of the World Online (POWO) serves as a taxonomic backbone for tracheophytes on iNaturalist. There are some tools used for automatic, semi-automatic and manual addition of new taxa and modification of the taxonomic information. Reasonable deviations from POWO could be accepted on iNaturalist by the curators after community discussions. The taxonomic opinion of an observer, if necessary, may be recorded in the description section of an individual observation.

Unfortunately, Russia lacks both a modern checklist of vascular plants and a standard flora. Therefore, we could assume that the project covers ca. 55% of the Russian plant diversity out of 12,500 species estimated by Kamelin (2007). That is quite a satisfactory figure since the Russian flora includes many species which require collection and proper identification of herbarium specimens (*Hieracium*, *Alchemilla*, *Crataegus*, some Poaceae and Cyperaceae etc.). There are also many rare endemics in hardly accessible mountain areas and quite a few insufficiently-known species recorded from scattered localities.

The list of the most recorded species of the project includes species which are widespread, easily recognisable and identifiable during all seasons (Table 9). These are mostly perennial herbs tolerant to intensive human activity, but also some common trees. Since the observations are concentrated in European Russia (Fig. 5), top-observed species of the project perfectly match the most common plants of temperate Europe, based on frequency of occurrences in the national grid mapping projects (Seregin 2011). The five most observed species have more than 5,000 observations each.

There are 1215 unique observations in the project. They include:

- 994 species,
- 19 nothospecies (hybrids),
- 133 heterotypic infraspecific taxa,
- 36 homotypic infraspecific taxa,
- 33 genera lacking chance for finer identification.

The following users have created the greatest number of unique species and nothospecies records: R.A. Murtazaliev (195 observations), V.S. Volkotrub (81), M.M. Mallaliev (40), S.A. Nesterova (32), A.I. Pyak (32), S.R. Mayorov (31), S.A. Svirin (27), I.N. Pospelov (25), Aleksandr L. Ebel (23), D.A. Bochkov (23), D.G. Chimitov (19), M.S. Knyazev (17), A.P. Seregin (17), E.S. Kashirina (16), A.V. Popov (13), O.A. Chernyagina (13), E.A. Razina (12) and N.S. Liksakova (12). Altogether, 224 observers were lucky to contribute at least one unique observation of a species or a hybrid.

To assess the regional representation of our data, we have compiled a table on the regional diversity of the Russian flora with necessary references (Table 10). The numbers of known species across the regions are not always perfectly comparable, since the authors of regional floras, guides and checklists used various species concepts which were either "splitters" or "lumpers". The overestimate for Volgograd Oblast (Sagalaev 2008) is especially notable.

Table 10 gives a good overview of plant diversity across the country with notable hotspots revealed earlier by Malyschev for standard areas (Malyshev 1975, Malyshev 1992):

1. Caucasus (Dagestan, Krasnodar Krai, Kabardino-Balkaria, North Ossetia, Chechen Republic, Stavropol Krai etc.);
2. Russian Far East (Primorsky Krai, Khabarovsk Krai);
3. Southern Siberia (Irkutsk Oblast, Altai Krai, part of Krasnoyarsk Krai etc.);
4. Crimea.

Additionally, the Caucasus is listed as the only biodiversity - and especially tracheophyte diversity - hotspot of global importance in Russia (Myers et al. 2000, Barthlott et al. 2007).

We present data on the taxonomic diversity of vascular plants within the regions (Table 11) using two indexes, i.e. (1) the number of the recorded species and (2) the number of the lowest-rank taxa of the taxonomic tree with "research grade". The second index includes varieties, subspecies, species and those genera which cannot be accurately identified to species rank by uploaded photos (for instance, *Alchemilla*, *Pilosella*, *Hieracium*, *Euphrasia* and some genera requiring specific features not always captured by observers, like *Melilotus* and *Epilobium* without flowers etc.). The number of the recorded species is more suitable for the further taxonomic analysis.

The community has observed the highest number of plant species in Dagestan (1,927 species), which is the richest region of Russia in terms of the number of known species (Table 10). However, the rich flora of Dagestan is still represented here by only 57.0% of known species (Murtazaliev 2016). It is followed by other six territories of the Russian Federation from the above mentioned plant diversity hotspots — the Caucasus (represented by Krasnodar Krai), the Crimean Peninsula (the Republic of Crimea and the City of Sevastopol), the southern part of the Russian Far East (Primorsky Krai) and the mountains of Southern Siberia (Altai Krai). Four well-represented regions of Central Russia (Kursk, Voronezh, Moscow and Bryansk Oblasts) form the next group with a good proportion of flora detection ranging from 56.3% to 81.8%.

If we consider the number of species known from each region, Kursk Oblast is the leader in terms of the proportion of observed species (81.8% out of 1,409 known species). Nizhny Novgorod Oblast, Bryansk Oblast, Kostroma Oblast and the City of Sevastopol also have over 70% of known species already represented on iNaturalist, although Nizhny Novgorod Oblast lacks a modern flora checklist, since the number of taxa for the region published by Bakka and Kiseleva (2008) is out of date.

In regional lists, 53 species are counted as the leaders of the scoreboards (Table 12). This list includes some notable invasive species like *Acer negundo* (a leader in five regions), *Heracleum sosnowskyi* (two regions), *Ambrosia artemisiifolia*, *Erigeron annuus*, *Hordeum jubatum*, *Impatiens glandulifera*, and *Lupinus polyphyllus* (one region each). Usually, this high performance of invasive alien species is a result of intentional recording in line with a regional assessment of aliens performed by the project members.

Conifers, which could be observed during the whole year, provide another example of the most observed species across regions. For instance, *Pinus sylvestris* is a top species in ten regions as well as *Larix gmelinii* (two regions), *Picea obovata*, *Pinus sibirica* and *Juniperus deltoides* (one region).

Other examples include common plants of meadows and urban lawns (e.g. *Achillea millefolium*, *Taraxacum* aggr. *officinale*, *Tanacetum vulgare*, *Artemisia vulgaris*, *Cirsium arvense*, *Cichorium intybus*), common tundra plants in Arctic regions (*Cornus suecica*, *Papaver pulvinatum*, *Rubus chamaemorus*), species abundant in the taiga zone (*Vaccinium vitis-idaea*, *Chamaenerion angustifolium*) or common plants of dry grasslands (*Tulipa suaveolens*, *Fragaria viridis*, *Cichorium intybus*). There are exceptional cases of over-recording of orchids (*Cypripedium macranthos*, *Dactylorhiza euxina*).

## Temporal coverage

### Notes

**Uploading date**

The project started on 9 Jan 2019 with 11,000 "research-grade" observations of the Russian flora. As of 8 Sep 2020, observations uploaded to iNaturalist in 2018 and earlier, account for only 1.4% of all the project data (Table 13). The number of observations uploaded in the eight months of 2020 exceeds threefold the data uploaded in 2019. The backlog of unidentified observations from 2019 is much smaller than the proportion of unprocessed records made in 2020.

**Observation date**

Many participants of the "Flora of Russia" project hold large photo archives and continue to post them on iNaturalist retrospectively. Therefore, at least 14.9% of the observations were made before 31 Dec 2018 (Table 14). Since the project requires a photo of the organism, the most important limiting factor of the temporal coverage is the time of spreading of digital cameras. Apparently, their appearance in Russia, judging by the data, is dated 2002-2003. Amongst the earlier observations, there are both scanned photographs and transparencies, as well as later photographs of preserved specimens.

We have analysed the data on the basis of dates of observation for 2019 (21.5% of all data on plants in Russia) and the eight months of 2020 from January to August (64.6%). Two graphs given below have the same scale bar.

**Figure 7.**
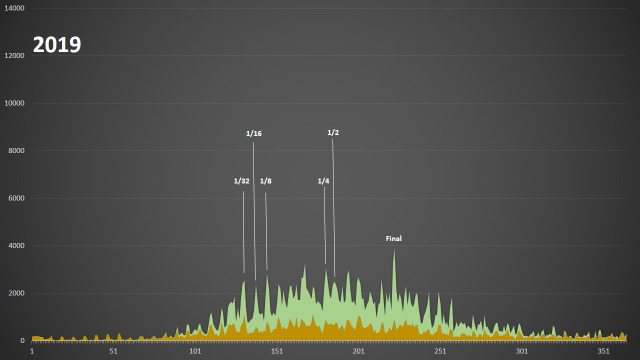
Verifiable observations from Russia on iNaturalist made in 2019 - tracheophytes (green) and all other groups (brown). A major event which contributed to data collection was the Russian Team Cup on photodocumentation of wild plants 2019 (from 1/32 to final).

In 2019, the most productive days were the first two days of the Team Cup final, when its participants made 3,027 (11 Aug 2019) and 2,602 (10 Aug 2019) observations of vascular plants (Fig. 7). This was mainly caused by the fact that we organised the final as a bioblitz, while in the early stages, it was possible to upload archived photos. However, the 2019 Cup overall did not attract much interest amongst the participants. For example, the third richest day by the number of observations was 17 Jun 2019, on which 2,514 observations of vascular plants were made, including 555 observations from the field trip of Lomonosov Moscow State University students to Voronezh Oblast.

**Figure 8.**
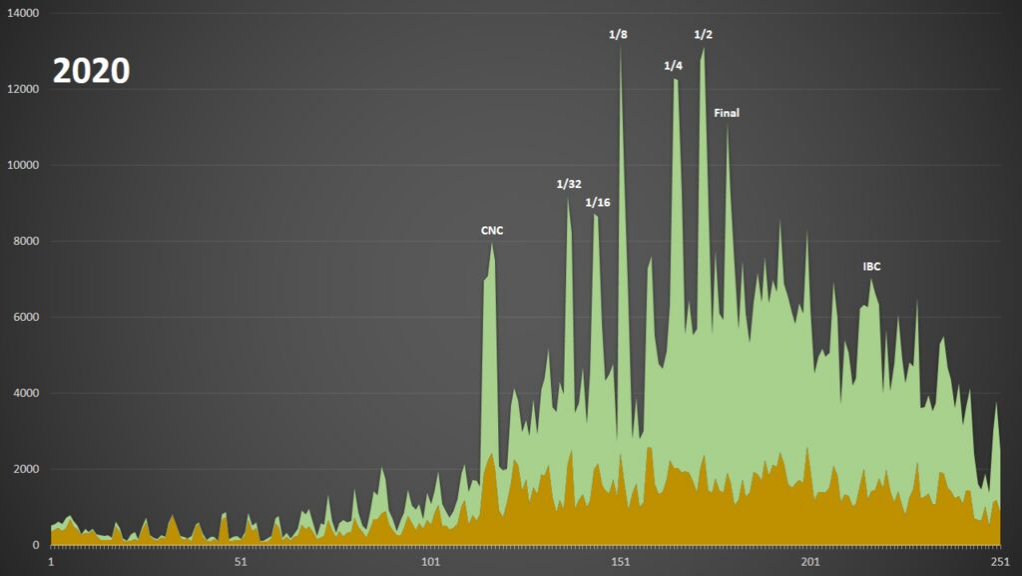
Verifiable observations from Russia on iNaturalist in 2020 - tracheophytes (green) and all other groups (brown). Major events contributed to data collection include the City Nature Challenge 2020 (CNC), the Russian Team Cup on photodocumentation of wild plants 2020 (from 1/32 to final) and the International Biodiversity Championship 2020 (IBC).

In 2020, on the contrary, all the six stages of the Cup are clearly visible as prominent peaks of observation numbers. Namely, on 30 May 2020, 10,780 observations of vascular plants were made during 1/8 of the Cup (16 teams) and during the first and second days of the semi-finals 10,724 and 10,734 observations were made by four regional teams (Fig. 8). From the 1/8 of the Cup onwards, the rounds were held in the format of a three-day bioblitz from Saturday to Monday.

International competitions like the City Nature Challenge (CNC) and the International Biodiversity Championship (IBC) did not generate peak user interest across Russia in 2020. However, both events also made a significant contribution to our data, since they lasted four days each. During the four CNC days (24-27 Apr 2020), 20,965 observations of vascular plants were made and 20,429 observations were recorded during the four IBC days (3-6 Aug 2020). We actively promoted both events amongst the participants of the "Flora of Russia" project.

It is worth mentioning that the COVID-19 restrictions of the spring of 2020 caused, for example, a low level of participation in CNC, which was made up for in the summer by off-campus student practices and events for high school students which all used iNaturalist this year.

Summing up, all Russian projects on student practices over the three summer months of 2020 (the common time for them in Russia) shows that 54,186 "research grade" observations by more than 750 observers meet the requirements of the "Flora of Russia" project. This makes a modest 17.4% contribution to the summer observations of the project. In 2020, practices in the form of independent work of students supervised remotely by teachers were held in fourteen Russian universities: Moscow State University, Bashkir State University, Irkutsk State University, State University of Nizhny Novgorod, Voronezh State University, Ural Federal University, Mordovia State University, Kazan Federal University, Tver State University, Kirov State University, Pushchino State Institute of Natural Science, Ivanovo State University and Tomsk State University.

Another notable income of the summer data flow was the Herbarium 2.0 project, organised by Valentina Borodulina. Being initially designed for high school students, it attracted the attention of schoolteachers and teachers of out-of-school education. Of the 44,087 observations of this project (1 Jun - 31 Aug 2020), 36,307 observations were made in Russia and reached "research-grade". This contributes to 11.0% of our summer data and the most active observers involved in the project rapidly became notable participants of the "Flora of Russia" project.

## Usage licence

### Usage licence

Other

## Data resources

### Data package title

Flora of Russia on iNaturalist backup 8 Sep 2020 (750K + 136K records)

### Resource link

http://doi.org/10.5281/zenodo.4061848, "Flora of Russia" backup (Zenodo), a stable snapshot of the dataset performed at 9 Sep 2020.

### Alternative identifiers

**Other endpoints for the same stable snapshot of the dataset performed at 9 Sep 2020**: https://zenodo.org/record/4061848#.X3afWe1n1PZ, "Flora of Russia" backup (alternative Zenodo identifier); https://doi.org/10.15468/ab3s5x, alternative identifier of complete iNaturalist dataset in GBIF; https://doi.org/10.13140/RG.2.2.17886.87362/1, "Flora of Russia" backup (Research Gate); https://www.researchgate.net/publication/344174058_Flora_of_Russia_on_iNaturalist_backup_2020_Sep_08_750K_136K_records, alternative Research Gate identifier. **Links to updated dynamic resources**: https://www.inaturalist.org/observations/export?projects=flora-of-russia, permanently updated csv-export of the "Flora of Russia" data (link to iNaturalist export tool); http://www.inaturalist.org/observations/gbif-observations-dwca.zip, complete iNaturalist dataset in GBIF (Ueda 2020); https://www.gbif.org/dataset/50c9509d-22c7-4a22-a47d-8c48425ef4a7, alternative identifier of complete iNaturalist dataset in GBIF.

### Number of data sets

1

### Data set 1.

#### Data set name

Flora of Russia on iNaturalist backup 8 Sep 2020 (750K + 136K records)

#### Data format

xlsx

#### Number of columns

28

#### Download URL

https://zenodo.org/record/4061848/files/flora-of-russia%26d1%3D1970-09-01%26d2%3D2019-06-30.xlsx?download=1; https://zenodo.org/record/4061848/files/flora-of-russia%26d1%3D2019-07-01%26d2%3D2020-05-20.xlsx?download=1; https://zenodo.org/record/4061848/files/flora-of-russia%26d1%3D2020-05-21%26d2%3D2020-07-05.xlsx?download=1; https://zenodo.org/record/4061848/files/flora-of-russia%26d1%3D2020-07-06%26d2%3D2020-09-08.xlsx?download=1; https://zenodo.org/record/4061848/files/flora-of-russia-needs-id-backlog.xlsx?download=1

#### Description

"Flora of Russia" on iNaturalist backup for 8 Sep 2020 (886K records in total - 750K confirmed photo observations on 6,857 species and additional 136K unverified photo observations). Contains metadata only and hyperlinks to photos in csv format. The backup was exported from iNaturalist.org using the "Export Observations" tool. We are using 27 columns for further processing out of 66 available columns, since the whole iNaturalist dataset in long-tailed.

We amended the dataset on 25 Sep 2020 after a data audit performed by Dr Robert Mesibov (https://www.datafix.com.au) in line with preparation of the data paper. All records with positional accuracy exceeding 50,000 m were marked as having inaccurate location and reported to users. Altogether, we excluded 1,106 observations from the project’s data and 587 observations from the backlog from the backup on this ground.

The “research-grade” observations with free licences (CC0, CC-BY and CC-BY-NC) are fully available in GBIF within “iNaturalist Research-grade Observations” occurrence dataset (https://doi.org/10.15468/ab3s5x). We added the last column "gbif_id" to all csv files of our dataset with URLs of GBIF records using GBIF Occurrence Download https://doi.org/10.15468/dl.msfxkn performed on 28 Sep 2020.

Five amended csv-files with 750,143 observations from the project “Flora of Russia” (“research-grade” records) and 136,023 observations the project’s backlog (“needs-id” records) represent the stable project backup (https://doi.org/10.5281/zenodo.4061848).

The following abbreviations are used in column descriptions:

- A - automatically generated data (usually from exif files of photos);
- M - manually inserted data;
- AM - both options are possible (automatically generated data which could be manually edited).

**Data set 1.** 

Column label	Column description
id	Unique identifier for the observation (A)
observed_on_string	Date/time as entered by the observer (AM)
observed_on	Normalised date of observation (A)
time_observed_at	Normalised date/time of observation (A)
time_zone	Time zone of observation (AM)
user_id	Unique identifier for the observer (A)
user_login	Username of the observer (A)
created_at	Date/time observation was created (A)
updated_at	Date/time observation was last updated (A)
quality_grade	Quality grade of this observation; "research grade" only for the "Flora of Russia" project and "needs ID" only for the project's backlog (A)
licence	Licence the observer has chosen for the media file supporting this observation (AM)
url	URL for the observation (A)
image_url	URL for the default image (A)
oauth_application_id	Which application was used to post the observation (A)
latitude	Publicly visible latitude (AM)
longitude	Publicly visible longitude (AM)
positional_accuracy	Accuracy estimate in metres (AM)
private_latitude	Private latitude, set if observation private or obscured (AM)
private_longitude	Private longitude, set if observation private or obscured (AM)
private_positional_accuracy	Coordinate precision, set if observation private or obscured (AM)
geoprivacy	Whether or not the observer has chosen to obscure or hide the coordinates (AM)
taxon_geoprivacy	Most conservative geoprivacy applied due to the conservation statuses of taxa in current identification (A)
coordinates_obscured	Whether or not the coordinates have been obscured, either because of geoprivacy or because of a threatened taxon (A)
positioning_device	Device used to determine coordinates (A)
positioning_method	How coordinates were determined (A)
scientific_name	Scientific name of the observed taxon according to iNaturalist taxonomic backbone (AM)
taxon_id	Unique identifier for the observed taxon (A)
gbif_id	URL for the corresponding GBIF record (A)

## Additional information

**Community Coverage**

**Number of observers.** The project reached a milestone of **10,000 observers** with at least a single "research grade" observation 7 Sep 2020.

The maximum number of observers is concentrated in the largest cities of Russia and their metropolitan areas - Moscow with Moscow Oblast and St. Petersburg with Leningrad Oblast (Table 15), followed by Krasnodar Krai and the Crimea, two resort regions on the Black Sea coast, which attract millions of tourists. Large communities have also formed in other major cities of Russia, for example, Nizhny Novgorod and Ekaterinburg (Sverdlovsk Oblast). Despite the geographic proximity of Moscow Oblast and the City of Moscow, they have two different communities, which overlap only by 33.4%. A similar situation is observed in St. Petersburg and Sevastopol. The communities of observers in St. Petersburg and Leningrad Oblast overlap by 37.0%, whereas in the Crimea and Sevastopol by 32.0%.

**Number of members (subscribers) of regional projects.** The largest regional community of formal members is in the City of Moscow (122 participants) and Moscow Oblast (88 participants). Membership in a regional project allows a member to follow news and to affiliate their observations with a specific region on the observation page. More than 40 participants joined the projects of Tula Oblast, Crimea, Novosibirsk Oblast, Bryansk Oblast, Krasnodar Krai, Sevastopol, Altai Krai and Sverdlovsk Oblast. In Kamchatka, 30.8% of observers are subscribers to the regional project, while in St. Petersburg, on the contrary, only 3.0% have subscribed to the regional project. The number of subscribers is a result of an active curation of the regional project journal.

**Number of observers per 1M capita.** The number of observers per 1M of the regional population shows how actively the local residents are involved in the work of the "Flora of Russia" project. However, a top list, with a few exceptions, includes regions with a small population and sites specifically noteworthy for naturalists. Due to tourist activity, a relatively high number of observers has been noted in Altai Republic, Kamchatka, Leningrad Oblast, Karelia, Chukotka, Nenets Autonomous Okrug and Kaluga Oblast. Communities mostly formed by local residents include Sevastopol, Moscow Oblast and Tver Oblast.

**Number of observers per 1,000 km^2^.** This index helps assess areas with a high density of observers. The federal cities of Moscow, St. Petersburg and Sevastopol are undoubtedly in the lead here (200-700 observers per 1,000 km^2^). This number is reduced to 43 observers in Moscow Oblast, followed by the Crimea (16), Tula Oblast (13) and Kaliningrad Oblast (11).

**Productivity (number of observations per observer).** This index clearly demonstrates the regions with a fairly modest community, where data are received mainly from a few of the most active participants ("mega-observers") (Table 16). Such active individuals greatly helped Omsk Oblast, Kursk Oblast, Kostroma Oblast, Bryansk Oblast, Chuvashia, Dagestan, Mordovia, Kamchatka, Sevastopol and Tyumen Oblast to rise high in the index.

**Data Usage**

The project's data were cited in a number of research papers dealing with documentation and verification of new regional records (Prokopenko et al. 2019; Verkhozina et al. 2019; Leostrin and Efimova 2020; Seregin 2020b; [Bibr B6121206][Bibr B6316170]).

Other examples of dataset usage include papers on distribution of noteworthy alien plants (Mayorov et al. 2020; Zarubo and Mayorov 2020), floristic inventories of protected areas (Seregin 2020a) and phenology of plants during the extremely warm winter of 2019/2020 (Vinogradov 2020).

Several papers on orchids of Russia employed our data to a various extent since this showy group attracts special attention of the observers (Efimov and Legchenko 2020; Efimov 2020; Popovich et al. 2020).

## Figures and Tables

**Figure 6a.**
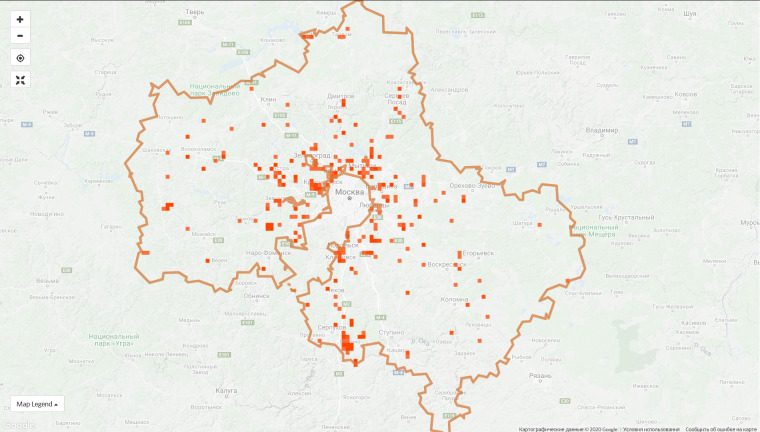
Moscow Oblast, 9 Jan 2019 - 1,286 observations from 180 users

**Figure 6b.**
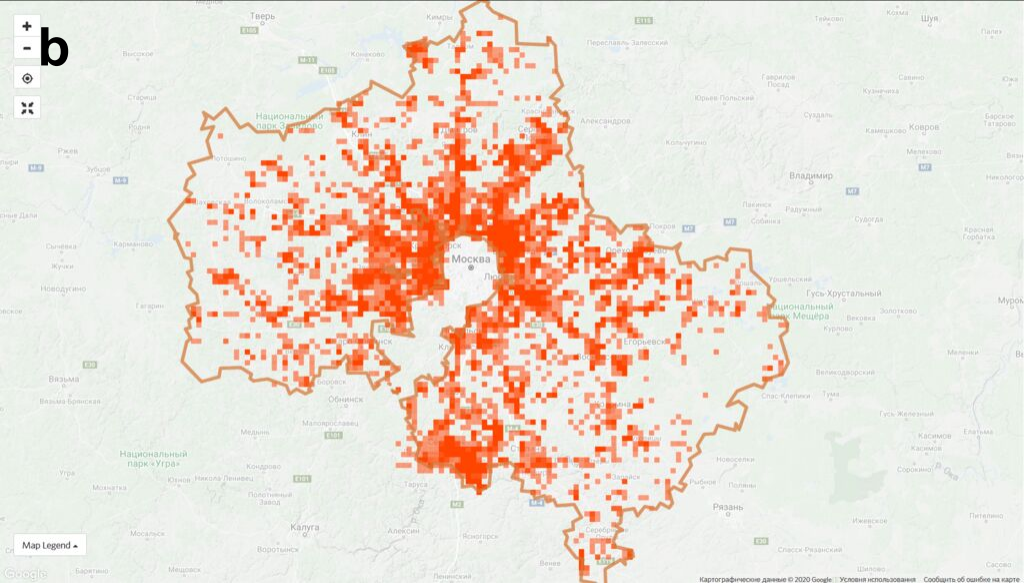
Moscow Oblast, 7 Sep 2020 - 72,861 observations from 1,906 users

**Figure 6c.**
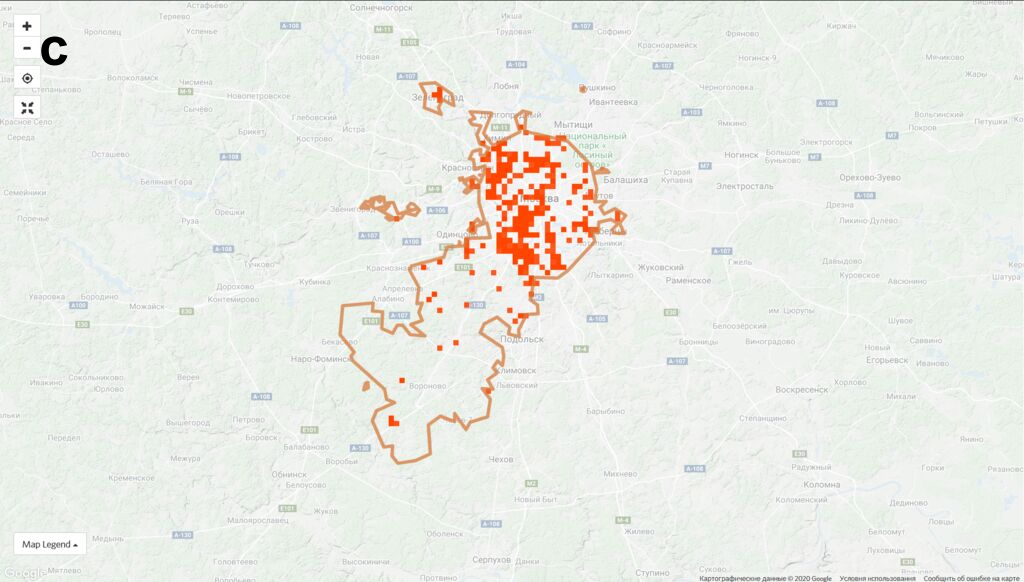
City of Moscow, 9 Jan 2019 - 1,115 observations from 181 users

**Figure 6d.**
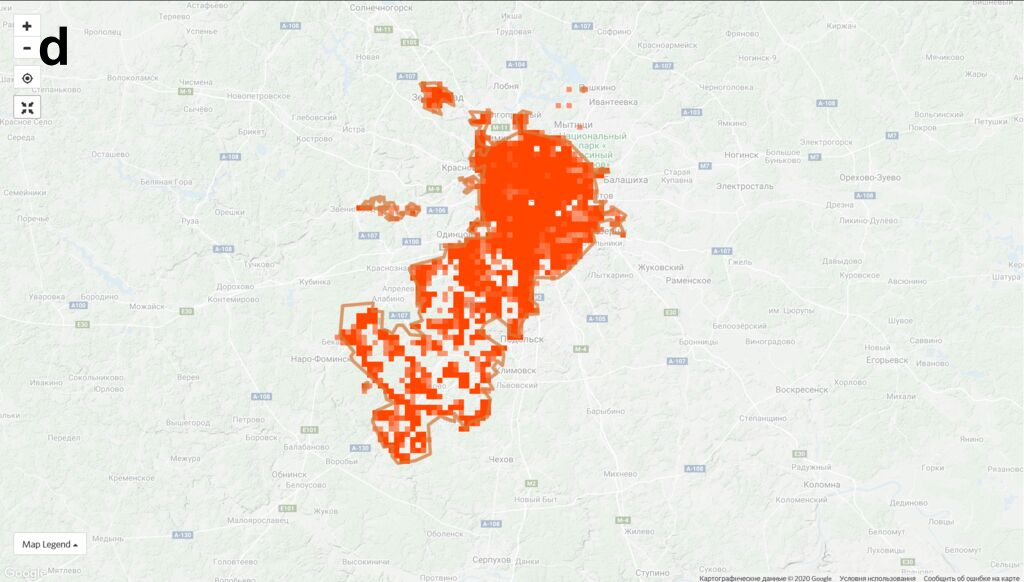
City of Moscow, 7 Sep 2020 - 66,124 observations from 1,596 users

**Figure 6e.**
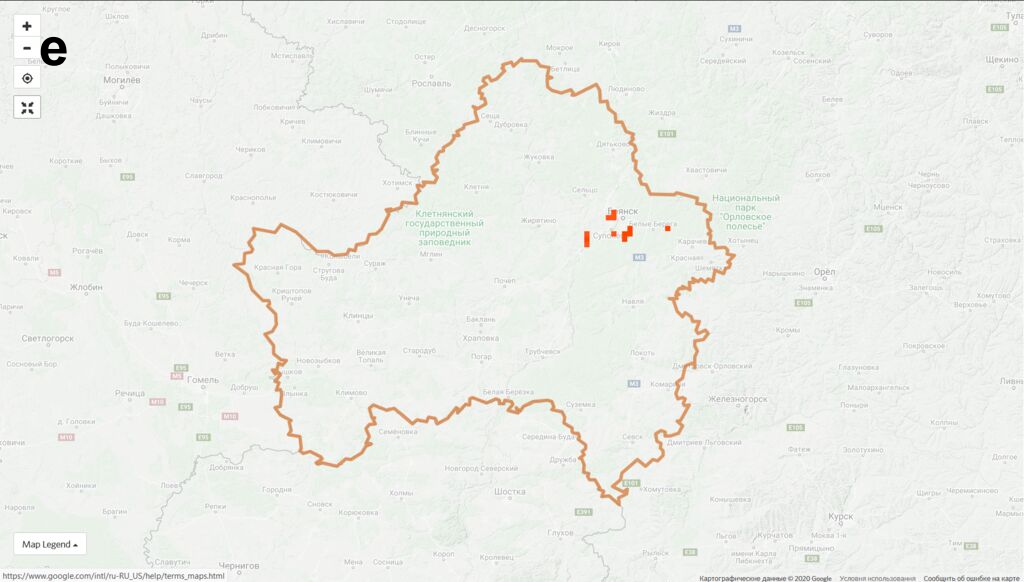
Bryansk Oblast, 9 Jan 2019 - 67 observations from 4 users

**Figure 6f.**
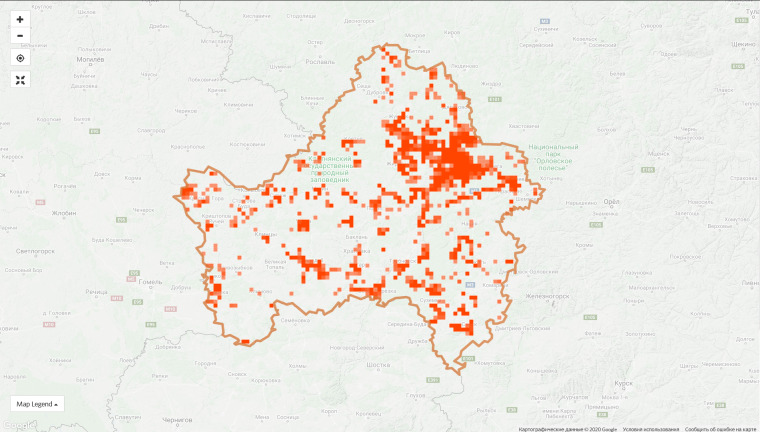
Bryansk Oblast, 7 Sep 2020 - 34,830 observations from 175 users

**Table 1.** The top ten countries by the number of verifiable observations on iNaturalist (as of 5 Sep 2020). In Tables 1-6 of this section, Russia is presented within the borders on 1 Jan 2014 (so called "standard places" on iNaturalist), i.e. excluding the Republic of Crimea and the City of Sevastopol claimed by Ukraine.

**Rank**	**Country**	**All groups**	**Tracheophytes**	**Proportion of tracheophytes amongst all groups**
1	USA	25,896,649	10,937,966	42.2
2	Canada	3,606,976	1,401,370	38.9
3	Mexico	2,305,831	721,168	31.3
4	Australia	1,366,788	298,223	21.8
5	Russia	1,329,399	826,949	62.2
6	UK	1,150,580	512,311	44.5
7	South Africa	972,158	548,997	56.5
8	New Zealand	821,308	376,942	45.9
9	Italy	705,192	243,265	34.5
10	Germany	689,644	247,961	36.0
	WORLD	48,612,707	19,308,096	39.7

**Table 2.** The proportion of popular taxonomic groups amongst verifiable observations for the top ten countries on iNaturalist (5 Sep 2020)

**Rank**	**Country**	**Tracheophytes**	**Birds**	**Insects**	**Other groups**
1	Russia	62	15	13	10
2	South Africa	56	11	15	18
3	New Zealand	46	11	17	27
4	UK	45	11	28	17
5	USA	42	13	24	22
6	Canada	39	13	28	20
7	Germany	36	15	34	15
8	Italy	34	12	34	20
9	Mexico	31	26	23	20
10	Australia	22	18	31	29
	WORLD	40	14	25	21

**Table 3.** Identified and verified ("research grade", RG) observations for the top ten countries on iNaturalist (as of 5 Sep 2020)

**Rank**	**Country**	**All groups**	**Tracheophytes**	**Proportion of tracheophytes in RG observations**	**Proportion of RG observations in tracheophytes**
1	USA	15,359,670	6,023,579	39.2	55.1
2	Canada	2,264,736	886,530	39.1	63.3
3	Mexico	1,472 829	437,017	29.7	60.6
4	Russia	1,049,298	704,273	67.1	85.2
5	Australia	864,251	173,741	20.1	58.3
6	UK	704,083	285,639	40.6	55.8
7	South Africa	630,269	360,377	57.2	65.6
8	New Zealand	589,106	311,365	52.9	82.6
9	Italy	459,868	142,800	31.1	58.7
10	Germany	455,542	171,487	37.6	69.2
	WORLD	29,184,780	11,091,055	38.0	57.4

**Table 4.** Verifiable observations with free licences (CC0, CC-BY & CC-BY-NC) for the top ten countries on iNaturalist (as of 5 Sep 2020)

**Rank**	**Country**	**All groups**	**Proportion of observations with free licences**	**Tracheophytes**	**Proportion of observations with free licences**
1	USA	15,566,609	60.1	6,568,148	60.0
2	Canada	2,483,532	68.9	965,480	68.9
3	Mexico	1,264,041	54.8	375,607	52.1
4	Russia	1,114,574	83.8	705,631	85.3
5	Australia	772,926	56.6	183,410	61.5
6	UK	729,997	63.4	322,184	62.9
7	South Africa	620,335	63.8	335,863	61.2
8	New Zealand	608,534	74.1	285,092	75.6
9	Germany	494,927	71.8	162,607	65.6
10	Italy	387,866	55.0	139,812	57.5
	WORLD	29,540,093	60.8	11,797,509	61.1

**Table 5.** iNaturalist records available in the GBIF ("research-grade" observations with free licences) for the top ten countries on iNaturalist (as of 3 Sep 2020) (Ueda 2020)

**Rank**	**Country**	**All groups**	**Proportion of GBIF records**	**Tracheophytes**	**Proportion of GBIF records**
1	USA	10,286,645	39.7	3,947,962	36.1
2	Canada	1,651,249	45.8	638,998	45.6
3	Russia	903,189	67.9	610,344	73.8
4	Mexico	897,372	38.9	241,058	33.4
5	Australia	688,655	50.4	129,962	43.6
6	New Zealand	495,567	60.3	262,461	69.6
7	UK	467,082	40.6	188,451	36.8
8	South Africa	447,995	46.1	250,086	45.6
9	Germany	354,619	51.4	128,778	51.9
10	Italy	289,076	41.0	107,780	44.3
	WORLD	19,745,698	40.6	7,409,526	38.4

**Table 6.** Observers and their average productivity for the top ten countries on iNaturalist (as of 5 Sep 2020)

**Rank**	**Country**	**All groups (observers with at least one verifiable observation)**	**Observations per observer**	**Tracheophytes (observers with at least one "research-grade" observation)**	**Observations per observer**
1	USA	706,531	37	321,154	19
2	Canada	87,559	41	40,415	22
3	Mexico	53,150	43	19,244	23
4	UK	49,955	23	22,228	13
5	Italy	25,331	28	8,839	16
6	Australia	23,679	58	7,098	24
7	Germany	17,278	40	7,691	22
8	New Zealand	16,535	50	7,095	44
9	Russia	14,328	93	9,602	73
10	South Africa	11,031	88	5,466	66
	WORLD	1,282,002	38	546,182	20

**Table 7.** Human population and area of the regions of Russia (official data)

**Regional project**	**Area**	**Rank (area)**	**Proportion in Russia's area**	**Population**	**Rank (population)**	**Proportion in Russia's population**	**Population density**	**Rank (population density)**
Flora of Yakutia	3,083,523	1	18.01	967,009	56	0.66	0.31	82
Krasnoyarsk Krai Flora	2,366,797	2	13.82	2,874,026	13	1.96	1.21	79
Khabarovsk Krai Flora	787,633	3	4.6	1,321,473	35	0.9	1.68	78
Irkutsk Oblast Flora	774,846	4	4.52	2,397,763	20	1.63	3.09	70
Flora of Yamalo-Nenets Autonomous Okrug	769,250	5	4.49	541,479	71	0.37	0.7	80
Flora of Chukotka	721,481	6	4.21	49,663	84	0.03	0.07	85
Flora of Khanty-Mansi Autonomous Okrug	534,801	7	3.12	1,663,795	28	1.13	3.11	69
Kamchatka Flora	464,275	8	2.71	314,723	79	0.21	0.68	81
Magadan Oblast Flora	462,464	9	2.7	141,234	83	0.1	0.31	83
Zabaykalsky Krai Flora	431,892	10	2.52	1,065,785	49	0.73	2.47	73
Komi Republic Flora	416,774	11	2.43	830,235	60	0.57	1.99	76
Arkhangelsk Oblast Flora	413,103	12	2.41	1,100,290	47	0.75	2.66	72
Amur Oblast Flora	361,908	13	2.11	793,194	62	0.54	2.19	75
Buryat Republic Flora	351,334	14	2.05	983,273	55	0.67	2.8	71
Tomsk Oblast Flora	314,391	15	1.84	1,077,442	48	0.73	3.43	67
Sverdlovsk Oblast Flora	194,307	16	1.13	4,315,699	5	2.94	22.21	43
Flora of Karelia	180,520	17	1.05	618,056	69	0.42	3.42	68
Novosibirsk Oblast Flora	177,756	18	1.04	2,793,384	15	1.9	15.71	50
Flora of Nenets Autonomous Okrug	176,810	19	1.03	43,829	85	0.03	0.25	84
Tyva Republic Flora	168,604	20	0.98	324,423	78	0.22	1.92	77
Altai Krai Flora	167,996	21	0.98	2,332,813	21	1.59	13.89	52
Primorsky Krai Flora	164,673	22	0.96	1,902,718	26	1.3	11.55	55
Perm Krai Flora	160,236	23	0.94	2,610,800	17	1.78	16.29	48
Tyumen Oblast Flora	160,122	24	0.94	1,518,695	30	1.03	9.48	60
Murmansk Oblast Flora	144,902	25	0.85	748,056	63	0.51	5.16	64
Vologda Oblast Flora	144,527	26	0.84	1,167,713	43	0.8	8.08	62
Bashkortostan Flora	142,947	27	0.83	4,051,005	7	2.76	28.34	39
Omsk Oblast Flora	141,140	28	0.82	1,944,195	24	1.32	13.77	53
Orenburg Oblast Flora	123,702	29	0.72	1,963,007	23	1.34	15.87	49
Kirov Oblast Flora	120,374	30	0.7	1,272,109	37	0.87	10.57	59
Volgograd Oblast Flora	112,877	31	0.66	2,507,509	18	1.71	22.21	44
Saratov Oblast Flora	101,240	32	0.59	2,440,815	19	1.66	24.11	42
Rostov Oblast Flora	100,967	33	0.59	4,202,320	6	2.86	41.62	25
Kemerovo Oblast Flora	95,725	34	0.56	2,674,256	16	1.82	27.94	41
Altai Republic Flora	92,903	35	0.54	218,866	81	0.15	2.36	74
Chelyabinsk Oblast Flora	88,529	36	0.52	3,475,753	9	2.37	39.26	26
Sakhalin Oblast Flora	87,101	37	0.51	489,638	74	0.33	5.62	63
Tver Oblast Flora	84,201	38	0.49	1,269,636	38	0.86	15.08	51
Leningrad Oblast Flora	83,908	39	0.49	1,847,867	27	1.26	22.02	45
Nizhny Novgorod Oblast Flora	76,624	40	0.45	3,214,623	10	2.19	41.95	24
Krasnodar Krai Flora	75,485	41	0.44	5,648,235	3	3.85	74.83	8
Flora of Kalmykia	74,731	42	0.44	272,647	80	0.19	3.65	66
Kurgan Oblast Flora	71,488	43	0.42	834,701	59	0.57	11.68	54
Tatarstan Flora	67,847	44	0.4	3,898,628	8	2.66	57.46	17
Stavropol Krai Flora	66,160	45	0.39	2,795,243	14	1.9	42.25	23
Flora of Khakassia	61,569	46	0.36	536,167	72	0.37	8.71	61
Kostroma Oblast Flora	60,211	47	0.35	637,267	67	0.43	10.58	58
Pskov Oblast Flora	55,399	48	0.32	629,651	68	0.43	11.37	56
Novgorod Oblast Flora	54,501	49	0.32	600,296	70	0.41	11.01	57
Samara Oblast Flora	53,565	50	0.31	3,183,038	11	2.17	59.42	14
Voronezh Oblast Flora	52,216	51	0.3	2,327,821	22	1.59	44.58	22
Dagestan Flora	50,270	52	0.29	3,086,126	12	2.1	61.39	13
Smolensk Oblast Flora	49,779	53	0.29	942,363	57	0.64	18.93	47
Astrakhan Oblast Flora	49,024	54	0.29	1,014,065	51	0.69	20.69	46
Moscow Oblast Flora	44,329	55	0.26	7,599,647	2	5.18	171.44	4
Penza Oblast Flora	43,352	56	0.25	1,318,103	36	0.9	30.4	35
Udmurt Republic Flora	42,061	57	0.25	1,507,390	31	1.03	35.84	28
Ryazan Oblast Flora	39,605	58	0.23	1,114,137	45	0.76	28.13	40
Ulyanovsk Oblast Flora	37,181	59	0.22	1,238,416	40	0.84	33.31	32
Flora of Jewish Autonomous Oblast	36,271	60	0.21	159,913	82	0.11	4.41	65
Yaroslavl Oblast Flora	36,177	61	0.21	1,259,612	39	0.86	34.82	29
Bryansk Oblast Flora	34,857	62	0.2	1,200,187	42	0.82	34.43	30
Tambov Oblast Flora	34,462	63	0.2	1,015,966	50	0.69	29.48	37
Kursk Oblast Flora	29,997	64	0.18	1,107,041	46	0.75	36.9	27
Kaluga Oblast Flora	29,777	65	0.17	1,009,377	52	0.69	33.9	31
Vladimir Oblast Flora	29,084	66	0.17	1,365,805	34	0.93	46.96	20
Belgorod Oblast Flora	27,134	67	0.16	1,547,418	29	1.05	57.03	18
Flora of Mordovia	26,128	68	0.15	795,504	61	0.54	30.45	34
Flora of the Crimea	26,081	69	0.15	1,911,818	25	1.3	73.3	9
Tula Oblast Flora	25,679	70	0.15	1,478,818	32	1.01	57.59	16
Oryol Oblast Flora	24,652	71	0.14	739,467	64	0.5	30	36
Lipetsk Oblast Flora	24,047	72	0.14	1,144,035	44	0.78	47.57	19
Mari El Flora	23,375	73	0.14	680,380	66	0.46	29.11	38
Ivanovo Oblast Flora	21,437	74	0.13	1,004,180	53	0.68	46.84	21
Chuvash Republic Flora	18,343	75	0.11	1,223,395	41	0.83	66.7	11
Chechen Republic Flora	15,647	76	0.09	1,456,951	33	0.99	93.11	6
Kaliningrad Oblast Flora	15,125	77	0.09	1,002,187	54	0.68	66.26	12
Flora of Karachay-Cherkessia	14,277	78	0.08	465,563	75	0.32	32.61	33
Flora of Kabardino-Balkaria	12,470	79	0.07	866,219	58	0.59	69.45	10
Flora of North Ossetia	7,987	80	0.05	699,253	65	0.48	87.55	7
Flora of Adygea	7,792	81	0.05	454,744	76	0.31	58.36	15
Flora of Ingushetia	3,628	82	0.02	497,393	73	0.34	137.1	5
Flora of Moscow	2,561	83	0.01	12,615,279	1	8.59	4,925.92	1
St Petersburg Flora	1,403	84	0.01	5,383,890	4	3.67	3,837.41	2
Sevastopol Flora	864	85	0.01	443,212	77	0.3	512.98	3

**Table 8.** Observations of the "Flora of Russia" project distributed amongst regional projects

**Regional project**	**Observa-tions**	**Rank (observa-tions)**	**Per 1K capita**	**Rank (per 1K capita)**	**Per 1,000 km^2^**	**Rank (per 1,000 km^2^)**	**Per recorded species**	**Rank (per recorded species)**
Moscow Oblast Flora	73,271	1	9.64	14	1,652.9	4	65.1	1
Flora of Moscow	66,227	2	5.25	29	25,859.8	1	60.9	2
Bryansk Oblast Flora	34,913	3	29.09	3	1,001.6	7	31.1	3
Tula Oblast Flora	27,456	4	18.57	8	1,069.2	5	29.0	4
Nizhny Novgorod Oblast Flora	26,667	5	8.30	17	348.0	14	26.5	5
Kursk Oblast Flora	26,340	6	23.79	4	878.1	8	22.8	8
Novosibirsk Oblast Flora	22,914	7	8.20	18	128.9	24	24.4	7
Sevastopol Flora	21,986	8	49.61	1	25,446.8	2	16.0	19
Altai Krai Flora	21,283	9	9.12	15	126.7	26	18.3	12
Omsk Oblast Flora	19,749	10	10.16	13	139.9	23	25.4	6
Irkutsk Oblast Flora	19,658	11	8.20	19	25.4	56	18.1	13
Sverdlovsk Oblast Flora	18,595	12	4.31	34	95.7	34	17.0	15
Chuvash Republic Flora	18,502	13	15.12	10	1,008.7	6	22.4	9
Tatarstan Flora	17,725	14	4.55	32	261.2	16	17.0	14
Voronezh Oblast Flora	17,401	15	7.48	22	333.3	15	15.4	20
Flora of Mordovia	16,654	16	20.94	5	637.4	9	19.7	10
Flora of the Crimea	16,562	17	8.66	16	635.0	10	10.5	31
Bashkortostan Flora	16,023	18	3.96	38	112.1	32	16.5	17
Yaroslavl Oblast Flora	15,091	19	11.98	11	417.1	13	18.4	11
Vladimir Oblast Flora	14,529	20	10.64	12	499.6	12	17.0	16
Kamchatka Flora	13,975	21	44.40	2	30.1	53	16.3	18
Kostroma Oblast Flora	12,728	22	19.97	6	211.4	19	15.0	21
Chelyabinsk Oblast Flora	10,214	23	2.94	45	115.4	29	14.2	23
Leningrad Oblast Flora	9,860	24	5.34	28	117.5	28	13.7	24
Tver Oblast Flora	9,607	25	7.57	21	114.1	30	12.7	26
St Petersburg Flora	9,164	26	1.70	60	6,531.7	3	14.7	22
Krasnodar Krai Flora	8,546	27	1.51	64	113.2	31	6.7	40
Samara Oblast Flora	8,520	28	2.68	48	159.1	21	11.1	29
Krasnoyarsk Krai Flora	8,395	29	2.92	46	3.5	74	7.7	37
Flora of Khanty-Mansi Autonomous Okrug	8,031	30	4.83	30	15.0	65	13.2	25
Primorsky Krai Flora	7,792	31	4.10	36	47.3	40	5.4	44
Kaliningrad Oblast Flora	7,700	32	7.68	20	509.1	11	11.2	27
Dagestan Flora	7,588	33	2.46	51	150.9	22	3.9	58
Kaluga Oblast Flora	7,404	34	7.34	23	248.6	17	10.9	30
Tyumen Oblast Flora	7,002	35	4.61	31	43.7	42	11.2	28
Belgorod Oblast Flora	6,271	36	4.05	37	231.1	18	8.1	34
Perm Krai Flora	5,832	37	2.23	53	36.4	47	8.5	32
Tomsk Oblast Flora	5,762	38	5.35	27	18.3	61	7.7	35
Kirov Oblast Flora	5,278	39	4.15	35	43.8	41	8.4	33
Udmurt Republic Flora	4,025	40	2.67	49	95.7	35	6.9	39
Buryat Republic Flora	3,644	41	3.71	40	10.4	68	4.3	52
Altai Republic Flora	3,571	42	16.32	9	38.4	45	4.6	50
Flora of Karelia	3,517	43	5.69	25	19.5	59	7.7	36
Volgograd Oblast Flora	3,512	44	1.40	65	31.1	51	4.7	49
Murmansk Oblast Flora	3,370	45	4.51	33	23.3	57	7.1	38
Saratov Oblast Flora	3,358	46	1.38	66	33.2	50	5.7	42
Ryazan Oblast Flora	3,319	47	2.98	44	83.8	36	5.8	41
Kemerovo Oblast Flora	3,305	48	1.24	67	34.5	48	5.0	45
Sakhalin Oblast Flora	3,198	49	6.53	24	36.7	46	4.2	54
Lipetsk Oblast Flora	3,087	50	2.70	47	128.4	25	5.6	43
Rostov Oblast Flora	2,920	51	0.69	73	28.9	54	3.7	59
Arkhangelsk Oblast Flora	2,822	52	2.56	50	6.8	73	4.0	57
Flora of Karachay-Cherkessia	2,642	53	5.67	26	185.1	20	3.6	60
Amur Oblast Flora	2,601	54	3.28	42	7.2	72	3.5	63
Ulyanovsk Oblast Flora	2,415	55	1.95	57	65.0	37	4.8	46
Pskov Oblast Flora	2,398	56	3.81	39	43.3	43	4.2	53
Ivanovo Oblast Flora	2,131	57	2.12	54	99.4	33	4.7	48
Penza Oblast Flora	2,067	58	1.57	62	47.7	39	3.6	61
Kurgan Oblast Flora	1,970	59	2.36	52	27.6	55	4.6	51
Flora of Yamalo-Nenets Autonomous Okrug	1,944	60	3.59	41	2.5	76	4.7	47
Vologda Oblast Flora	1,943	61	1.66	61	13.4	66	4.1	56
Novgorod Oblast Flora	1,832	62	3.05	43	33.6	49	4.1	55
Mari El Flora	1,207	63	1.77	59	51.6	38	2.7	67
Orenburg Oblast Flora	1,179	64	0.60	76	9.5	69	2.7	66
Stavropol Krai Flora	1,149	65	0.41	81	17.4	62	2.6	68
Flora of Khakassia	1,135	66	2.12	56	18.4	60	2.8	65
Tambov Oblast Flora	1,069	67	1.05	68	31.0	52	3.6	62
Zabaykalsky Krai Flora	1,024	68	0.96	69	2.4	77	2.3	72
Flora of Adygea	965	69	2.12	55	123.8	27	2.1	74
Flora of Chukotka	928	70	18.69	7	1.3	79	3.3	64
Smolensk Oblast Flora	856	71	0.91	70	17.2	63	2.5	70
Komi Republic Flora	739	72	0.89	71	1.8	78	2.4	71
Khabarovsk Krai Flora	609	73	0.46	79	0.8	81	2.0	75
Oryol Oblast Flora	570	74	0.77	72	23.1	58	2.2	73
Flora of Yakutia	525	75	0.54	78	0.2	85	1.7	78
Flora of Kabardino-Balkaria	487	76	0.56	77	39.1	44	1.8	77
Astrakhan Oblast Flora	467	77	0.46	80	9.5	70	2.5	69
Magadan Oblast Flora	221	78	1.56	63	0.5	83	1.9	76
Tyva Republic Flora	205	79	0.63	75	1.2	80	1.3	83
Chechen Republic Flora	175	80	0.12	84	11.2	67	1.3	82
Flora of North Ossetia	128	81	0.18	82	16.0	64	1.2	84
Flora of Jewish Autonomous Oblast	110	82	0.69	74	3.0	75	1.6	80
Flora of Nenets Autonomous Okrug	81	83	1.85	58	0.5	84	1.5	81
Flora of Kalmykia	48	84	0.18	83	0.6	82	1.6	79
Flora of Ingushetia	32	85	0.06	85	8.8	71	1.0	85

**Table 9.** The top 20 species of the "Flora of Russia" project ordered by the number of observations

**Rank**	Species	Number of observations
1	*Urtica dioica*	5,788
2	*Achillea millefolium*	5,536
3	*Pinus sylvestris*	5,375
4	*Taraxacum* aggr. *officinale*	5,327
5	*Cirsium arvense*	4,994
6	*Acer negundo*	4,874
7	*Tanacetum vulgare*	4,417
8	*Artemisia vulgaris*	4,404
9	*Trifolium pratense*	4,392
10	*Tussilago farfara*	4,293
11	*Chelidonium majus*	4,213
12	*Tripleurospermum inodorum*	4,183
13	*Plantago major*	4,184
14	*Cichorium intybus*	4,135
15	*Chamaenerion angustifolium*	4,109
16	*Trifolium repens*	3,797
17	*Sorbus aucuparia*	3,751
18	*Glechoma hederacea*	3,750
19	*Aegopodium podagraria*	3,727
20	*Veronica chamaedrys*	3,530

**Table 10.** Number of known species across the first-level administrative units of Russia with references.

**Rank**	**Regional project**	**Number of known species**	**Reference**
1	Dagestan Flora	3,380	Murtazaliev (2016)
2	Volgograd Oblast Flora	2,970	Sagalaev (2008), overestimate
3	Primorsky Krai Flora	2,750	Kozhevnikov and Kozhevnikova (2014)
4	Krasnodar Krai Flora	2,600	Zernov (2006)
5	Flora of the Crimea	2,573	Yena (2018)
6	Khabarovsk Krai Flora	2,516	Shlotgauer et al. (2001)
7	Flora of Kabardino-Balkaria	2,350	Shkhagapsoev (2015)
8	Flora of North Ossetia	2,306	Komzha (2000)
9	Irkutsk Oblast Flora	2,295	Chepinoga et al. (2008)
10	Chechen Republic Flora	2,295	Taysumov and Omarkhadzhieva (2012)
11	Altai Krai Flora	2,264	Silantieva (2013)
12	Stavropol Krai Flora	2,257	Ivanov (2005)
13	Krasnoyarsk Krai Flora	2,200	counts based on Krasnoborov (1988), Krasnoborov (1997), Krasnoborov and Malyshev (1988), Malyshev (1997), Malyshev and Peshkova (1987), Malyshev and Peshkova (1990), Malyshev and Peshkova (1993a), Malyshev and Peshkova (1993b), Malyshev et al. (2003), Peshkova (1996), Peshkova and Malyshev (1990), Polozhy and Malyshev (1994a), Polozhy and Malyshev (1994b), Polozhy and Peshkova (1996)
14	Buryat Republic Flora	2,161	Anenkhonov (2001)
15	Altai Republic Flora	2,136	Krasnoborov and Artemov (2012)
16	Udmurt Republic Flora	2,073	Baranova and Puzyrev (2012)
17	Tyva Republic Flora	2,066	Shaulo (2007)
18	Amur Oblast Flora	2,024	Starchenko (2008)
19	Sakhalin Oblast Flora	2,000	Eremin (2005)
20	Moscow Oblast Flora	2,000	Varlygina et al. (2008)
21	Flora of Karachay-Cherkessia	2,000	Zernov et al. (2015)
22	Flora of Adygea	2,000	Zamotailov (2012)
23	Flora of Yakutia	1,987	Kuznetsova and Zakharova (2012)
24	Rostov Oblast Flora	1,982	Fedyaeva (2014)
25	Voronezh Oblast Flora	1,954	A.V. Shcherbakov (personal communication)
26	Flora of Moscow	1,908	Shcherbakov and Lyubeznova (2018)
27	Samara Oblast Flora	1,900	Senator and Saksonov (2017)
28	Orenburg Oblast Flora	1,870	Ryabinina and Knyazev (2009); M.S. Knyazev (personal communication)
29	Sevastopol Flora	1,859	Seregin et al. (2015)
30	Flora of Khakassia	1,850	Aleksandr L. Ebel (personal communication)
31	Flora of Karelia	1,814	Kravchenko (2007)
32	Tver Oblast Flora	1,798	Notov et al. (2014)
33	Ulyanovsk Oblast Flora	1,760	Rakov et al. (2014)
34	Kemerovo Oblast Flora	1,753	Sheremetova et al. (2011)
35	Bashkortostan Flora	1,730	Naumova et al. (2011)
36	Sverdlovsk Oblast Flora	1,715	Knyazev et al. (2019)
37	Penza Oblast Flora	1,700	Vasyukov and Saksonov (2020)
38	Zabaykalsky Krai Flora	1,700	Popova (2017)
39	Chelyabinsk Oblast Flora	1,680	Kulikov (2005)
40	Belgorod Oblast Flora	1,680	N.M. Reshetnikova (personal communication)
41	Lipetsk Oblast Flora	1,669	A.V. Shcherbakov (personal communication)
42	Perm Krai Flora	1,658	Ovesnov (2007)
43	Tatarstan Flora	1,610	Bakin et al. (2000)
44	Tambov Oblast Flora	1,605	A.V. Shcherbakov (personal communication) (1478 species recorded by Sukhorukov (2010))
45	Oryol Oblast Flora	1,605	Kiseleva et al. (2012)
46	Leningrad Oblast Flora	1,600	Tzvelev (2000)
47	Chuvash Republic Flora	1,586	Gafurova (2014)
48	Kaluga Oblast Flora	1,542	Reshetnikova (2016)
49	Flora of Ingushetia	1,531	Dakieva (2003)
50	Yaroslavl Oblast Flora	1,500	estimate (previous number of species by Tikhomirov (1986) is out of date)
51	Saratov Oblast Flora	1,492	Bulany (2010)
52	Ryazan Oblast Flora	1,475	Kazakova and Shcherbakov (2017)
53	Kirov Oblast Flora	1,470	Tarasova (2007)
54	Tula Oblast Flora	1,465	A.V. Shcherbakov (personal communication)
55	Magadan Oblast Flora	1,457	Berkutenko (2010)
56	Bryansk Oblast Flora	1,451	Bulokhov et al. (2005)
57	Vologda Oblast Flora	1,450	Konechnaya and Suslova (2004)
58	Flora of Jewish Autonomous Oblast	1,443	Rubtsova (2019)
59	Kaliningrad Oblast Flora	1,436	Gubareva et al. (1999)
60	Ivanovo Oblast Flora	1,418	A.V. Shcherbakov (personal communication)
61	Kursk Oblast Flora	1,409	Poluyanov (2005)
62	Flora of Mordovia	1,401	Silaeva et al. (2010)
63	Vladimir Oblast Flora	1,399	Seregin (2014)
64	Tyumen Oblast Flora	1,395	Kuzmin (2018)
65	Novosibirsk Oblast Flora	1,379	Klescheva (2011)
66	Murmansk Oblast Flora	1,357	Konstantinova et al. (2014)
67	Smolensk Oblast Flora	1,310	Reshetnikova (2004); N.M. Reshetnikova (personal communication)
68	Kamchatka Flora	1,300	Chernyagina (2018)
69	Kurgan Oblast Flora	1,300	Bolshakov (2012)
70	Nizhny Novgorod Oblast Flora	1,290	Bakka and Kiseleva (2008)
71	Mari El Flora	1,259	Abramov (1995)
72	Astrakhan Oblast Flora	1,253	Laktionov (2009)
73	Pskov Oblast Flora	1,248	Konechnaya and Efimov (2018)
74	Flora of Khanty-Mansi Autonomous Okrug	1,175	Nazarenko and Pasechnyuk (2019) based on monograph by Krasnoborov (2006)
75	Novgorod Oblast Flora	1,174	Yurova et al. (2009)
76	Tomsk Oblast Flora	1,170	Revushkin (2014)
77	Omsk Oblast Flora	1,161	Bekisheva (1999); A.N. Efremov (personal communication)
78	Komi Republic Flora	1,158	Taskaev (2009)
79	Kostroma Oblast Flora	1,130	Leostrin and Efimova (2018)
80	Arkhangelsk Oblast Flora	1,098	Schmidt (2005)
81	St Petersburg Flora	1,088	Budantsev and Yakovlev (2006)
82	Flora of Yamalo-Nenets Autonomous Okrug	1,073	Pismarkina (2019)
83	Flora of Kalmykia	994	Baktasheva (2012)
84	Flora of Chukotka	936	Yurtsev et al. (2010)
85	Flora of Nenets Autonomous Okrug	720	Matveeva (2006)

**Table 11.** Taxonomic diversity of the "Flora of Russia" project across regional projects (references for species known in the region are given in Table 10)

**Regional project**	**Recorded species**	**Rank (recorded species)**	**Recorded taxa of the lowest rank**	**Species known in the region**	**Proportion of recorded species**	**Rank (proportion of recorded species)**
Dagestan Flora	1,927	1	1,960	3,380	57.0	19
Flora of the Crimea	1,570	2	1,643	2,573	61.0	15
Primorsky Krai Flora	1,430	3	1,491	2,750	52.0	25
Sevastopol Flora	1,378	4	1,405	1,859	74.1	5
Krasnodar Krai Flora	1,278	5	1,302	2,600	49.2	29
Altai Krai Flora	1,161	6	1,217	2,264	51.3	27
Kursk Oblast Flora	1,153	7	1,185	1,409	81.8	1
Voronezh Oblast Flora	1,131	8	1,187	1,954	57.9	17
Moscow Oblast Flora	1,126	9	1,213	2,000	56.3	21
Bryansk Oblast Flora	1,121	10	1,214	1,451	77.3	3
Sverdlovsk Oblast Flora	1,095	11	1,146	1,715	63.8	12
Krasnoyarsk Krai Flora	1,094	12	1,131	2,200	49.7	28
Irkutsk Oblast Flora	1,089	13	1,130	2,295	47.5	31
Flora of Moscow	1,087	14	1,176	1,908	57.0	20
Tatarstan Flora	1,041	15	1,074	1,610	64.7	11
Nizhny Novgorod Oblast Flora	1,006	16	1,008	1,290	78.0	2
Bashkortostan Flora	970	17	1,014	1,730	56.1	22
Tula Oblast Flora	948	18	991	1,465	64.7	10
Novosibirsk Oblast Flora	940	19	975	1,379	68.2	6
Vladimir Oblast Flora	856	20	907	1,399	61.2	14
Kamchatka Flora	856	21	898	1,300	65.8	8
Kostroma Oblast Flora	846	22	898	1,130	74.9	4
Flora of Mordovia	845	23	844	1,401	60.3	16
Buryat Republic Flora	838	24	852	2,161	38.8	45
Chuvash Republic Flora	827	25	850	1,586	52.1	24
Yaroslavl Oblast Flora	818	26	876	1,500	54.5	23
Rostov Oblast Flora	797	27	820	1,982	40.2	42
Omsk Oblast Flora	777	28	814	1,161	66.9	7
Altai Republic Flora	777	29	802	2,136	36.4	52
Belgorod Oblast Flora	776	30	808	1,680	46.2	32
Samara Oblast Flora	769	31	795	1,900	40.5	41
Tver Oblast Flora	757	32	797	1,798	42.1	39
Sakhalin Oblast Flora	755	33	771	2,000	37.8	49
Volgograd Oblast Flora	744	34	747	2,970	25.1	67
Tomsk Oblast Flora	744	35	768	1,170	63.6	13
Amur Oblast Flora	737	36	765	2,024	36.4	51
Flora of Karachay-Cherkessia	731	37	753	2,000	36.6	50
Chelyabinsk Oblast Flora	720	38	738	1,680	42.9	37
Leningrad Oblast Flora	718	39	714	1,600	44.9	34
Arkhangelsk Oblast Flora	714	40	728	1,098	65.0	9
Perm Krai Flora	689	41	702	1,658	41.6	40
Kaliningrad Oblast Flora	685	42	714	1,436	47.7	30
Kaluga Oblast Flora	678	43	708	1,542	44.0	36
Kemerovo Oblast Flora	665	44	695	1,753	37.9	47
Kirov Oblast Flora	628	45	654	1,470	42.7	38
Tyumen Oblast Flora	625	46	642	1,395	44.8	35
St Petersburg Flora	624	47	623	1,088	57.4	18
Flora of Khanty-Mansi Autonomous Okrug	610	48	631	1,175	51.9	26
Saratov Oblast Flora	591	49	601	1,492	39.6	43
Udmurt Republic Flora	581	50	595	2,073	28.0	62
Penza Oblast Flora	579	51	593	1,700	34.1	55
Ryazan Oblast Flora	575	52	591	1,475	39.0	44
Pskov Oblast Flora	565	53	578	1,248	45.3	33
Lipetsk Oblast Flora	554	54	568	1,669	33.2	56
Ulyanovsk Oblast Flora	503	55	516	1,760	28.6	61
Murmansk Oblast Flora	475	56	497	1,357	35.0	54
Vologda Oblast Flora	472	57	486	1,450	32.6	58
Flora of Karelia	457	58	474	1,814	25.2	66
Flora of Adygea	453	59	454	2,000	22.7	69
Ivanovo Oblast Flora	451	60	461	1,418	31.8	59
Mari El Flora	450	61	459	1,259	35.7	53
Zabaykalsky Krai Flora	449	62	455	1,700	26.4	63
Novgorod Oblast Flora	445	63	459	1,174	37.9	48
Stavropol Krai Flora	441	64	444	2,257	19.5	71
Orenburg Oblast Flora	434	65	439	1,870	23.2	68
Kurgan Oblast Flora	430	66	436	1,300	33.1	57
Flora of Yamalo-Nenets Autonomous Okrug	410	67	424	1,073	38.2	46
Flora of Khakassia	401	68	417	1,850	21.7	70
Smolensk Oblast Flora	343	69	355	1,310	26.2	64
Flora of Yakutia	303	70	305	1,987	15.2	74
Komi Republic Flora	302	71	304	1,158	26.1	65
Tambov Oblast Flora	300	72	308	1,605	18.7	72
Khabarovsk Krai Flora	299	73	301	2,516	11.9	76
Flora of Chukotka	285	74	289	936	30.4	60
Flora of Kabardino-Balkaria	268	75	271	2,350	11.4	77
Oryol Oblast Flora	258	76	255	1,605	16.1	73
Astrakhan Oblast Flora	187	77	190	1,253	14.9	75
Tyva Republic Flora	164	78	165	2,066	7.9	78
Chechen Republic Flora	140	79	140	2,295	6.1	81
Magadan Oblast Flora	114	80	114	1,457	7.8	79
Flora of North Ossetia	111	81	111	2,306	4.8	83
Flora of Jewish Autonomous Oblast	70	82	70	1,443	4.9	82
Flora of Nenets Autonomous Okrug	54	83	55	720	7.5	80
Flora of Ingushetia	31	84	31	1,531	2.0	85
Flora of Kalmykia	30	85	30	994	3.0	84

**Table 12.** The most recorded species in the regional projects (as of 9-10 Sep 2020)

**Species**	**Number of observations in the regional project**	**Regional project**
*Acer negundo*	109	Tyumen Oblast Flora
*Acer negundo*	174	Tomsk Oblast Flora
*Acer negundo*	279	Altai Krai Flora
*Acer negundo*	399	Bryansk Oblast Flora
*Acer negundo*	633	Tatarstan Flora
*Achillea millefolium*	3	Chechen Republic Flora
*Achillea millefolium*	27	Penza Oblast Flora
*Achillea millefolium*	41	Saratov Oblast Flora
*Achillea millefolium*	192	Bashkortostan Flora
*Achillea millefolium*	265	Nizhny Novgorod Oblast Flora
*Ambrosia artemisiifolia*	19	Stavropol Krai Flora
*Artemisia vulgaris*	116	Flora of Khanty-Mansi Autonomous Okrug
*Asplenium scolopendrium*	75	Krasnodar Krai Flora
*Betonica macrantha*	18	Flora of Adygea
*Campanula patula*	35	Udmurt Republic Flora
*Centaurea scabiosa*	60	Perm Krai Flora
*Chamaenerion angustifolium*	10	Flora of Yakutia
*Chamaenerion angustifolium*	155	Sverdlovsk Oblast Flora
*Chamaenerion angustifolium*	177	Leningrad Oblast Flora
*Chelidonium majus*	43	Tambov Oblast Flora
*Cichorium intybus*	19	Orenburg Oblast Flora
*Cichorium intybus*	34	Volgograd Oblast Flora
*Cirsium arvense*	162	Chelyabinsk Oblast Flora
*Cornus suecica*	7	Magadan Oblast Flora
*Cornus suecica*	80	Murmansk Oblast Flora
*Cypripedium macranthos*	308	Novosibirsk Oblast Flora
*Dactylorhiza euxina*	3	Flora of North Ossetia
*Delphinium grandiflorum*	19	Flora of Khakassia
*Diplotaxis tenuifolia*	117	Flora of the Crimea
*Echium vulgare*	29	Rostov Oblast Flora
*Echium vulgare*	166	Voronezh Oblast Flora
*Erigeron annuus*	11	Flora of Kabardino-Balkaria
*Erythronium sibiricum*	28	Kemerovo Oblast Flora
*Fragaria viridis*	27	Kurgan Oblast Flora
*Fritillaria camschatcensis*	149	Kamchatka Flora
*Gentiana algida*	5	Tyva Republic Flora
*Gentiana septemfida*	25	Flora of Karachay-Cherkessia
*Heracleum sosnowskyi*	312	Tver Oblast Flora
*Heracleum sosnowskyi*	304	Kursk Oblast Flora
*Hordeum jubatum*	195	Irkutsk Oblast Flora
*Impatiens glandulifera*	30	Primorsky Krai Flora
*Juniperus deltoides*	154	Sevastopol Flora
*Larix gmelinii*	51	Amur Oblast Flora
*Larix gmelinii*	44	Sakhalin Oblast Flora
*Leonurus quinquelobatus*	775	Moscow Oblast Flora
*Lupinus polyphyllus*	73	Kaliningrad Oblast Flora
*Melampyrum nemorosum*	74	Kaluga Oblast Flora
*Orostachys spinosa*	67	Altai Republic Flora
*Oxytropis myriophylla*	15	Zabaykalsky Krai Flora
*Papaver pulvinatum*	124	Krasnoyarsk Krai Flora
*Phragmites australis*	14	Astrakhan Oblast Flora
*Picea obovata*	25	Komi Republic Flora
*Pinus sibirica*	445	Omsk Oblast Flora
*Pinus sylvestris*	2	Flora of Ingushetia
*Pinus sylvestris*	12	Mari El Flora
*Pinus sylvestris*	24	Pskov Oblast Flora
*Pinus sylvestris*	26	Dagestan Flora
*Pinus sylvestris*	26	Novgorod Oblast Flora
*Pinus sylvestris*	31	Ivanovo Oblast Flora
*Pinus sylvestris*	95	Flora of Karelia
*Pinus sylvestris*	103	Ulyanovsk Oblast Flora
*Pinus sylvestris*	293	Vladimir Oblast Flora
*Pinus sylvestris*	293	Flora of Mordovia
*Populus tremula*	3	Flora of Jewish Autonomous Oblast
*Rhodiola rosea*	5	Flora of Nenets Autonomous Okrug
*Rhodiola rosea*	16	Flora of Chukotka
*Rubus chamaemorus*	54	Flora of Yamalo-Nenets Autonomous Okrug
*Salix myrsinifolia*	80	Arkhangelsk Oblast Flora
*Securigera varia*	66	Belgorod Oblast Flora
*Spiraea salicifolia*	14	Khabarovsk Krai Flora
*Tanacetum vulgare*	50	Lipetsk Oblast Flora
*Tanacetum vulgare*	122	Kostroma Oblast Flora
*Taraxacum* aggr. *officinale*	122	Samara Oblast Flora
*Taraxacum* aggr. *officinale*	169	St Petersburg Flora
*Taraxacum* aggr. *officinale*	1,064	Flora of Moscow
*Trifolium repens*	11	Oryol Oblast Flora
*Tulipa suaveolens*	7	Flora of Kalmykia
*Tussilago farfara*	86	Ryazan Oblast Flora
*Urtica dioica*	28	Vologda Oblast Flora
*Urtica dioica*	187	Yaroslavl Oblast Flora
*Urtica dioica*	204	Chuvash Republic Flora
*Urtica dioica*	223	Tula Oblast Flora
*Vaccinium vitis-idaea*	24	Buryat Republic Flora
*Vaccinium vitis-idaea*	59	Kirov Oblast Flora
*Veronica chamaedrys*	13	Smolensk Oblast Flora

**Table 13.** Observations of vascular plants of Russia by the year of uploading (as of 7 Sep 2020)

**Year**	"**Research grade" observations**	**Needs ID observations**	**All verifiable observations**	"**Research grade" (%)**	**Needs ID (%)**	**All verifiable (%)**
2018 and before	10,841	607	11,448	1.4	0.4	1.3
2019	212,662	19,075	231,737	28.3	14.0	26.1
2020	527,713	116,862	644,575	70.2	85.6	72.6
TOTAL	751,216	136,544	887,760	100.0	100.0	100.0

**Table 14.** Observations of vascular plants of Russia ordered by the year of record (as of 7 Sep 2020)

**Year**	"**Research grade" observations**	**Needs ID observations**	**All verifiable observations**	"**Research grade" (%)**	**Needs ID (%)**	**All verifiable (%)**
2001 and before	642	110	752	0.0	0.0	0.0
2002	341	24	365	0.0	0.0	0.0
2003	662	52	714	0.1	0.0	0.1
2004	1,011	96	1,107	0.1	0.1	0.1
2005	2,146	161	2,307	0.3	0.1	0.3
2006	2,570	199	2,769	0.3	0.1	0.3
2007	4,625	528	5,153	0.6	0.4	0.6
2008	5,803	451	6,254	0.8	0.3	0.7
2009	5,637	548	6,185	0.8	0.4	0.7
2010	7,967	1,603	9,570	1.1	1.2	1.1
2011	6,029	600	6,629	0.8	0.4	0.7
2012	7,059	793	7,852	0.9	0.6	0.9
2013	8,262	875	9,137	1.1	0.6	1.0
2014	6,371	649	7,020	0.8	0.5	0.8
2015	9,576	822	10,398	1.3	0.6	1.2
2016	11,404	1,008	12,412	1.5	0.7	1.4
2017	13,622	1,101	14,723	1.8	0.8	1.7
2018	18,490	2,028	20,518	2.5	1.5	2.3
2019	173,247	17,496	190,743	23.1	12.8	21.5
2020	465,752	107,400	573,152	62.0	78.7	64.6
TOTAL	751,216	136,544	887,760	100.0	100.0	100.0

**Table 15.** Community of the "Flora of Russia" project across regional projects

**Regional project**	**Obser-vers**	**Rank (obser-vers)**	**Regi-onal project mem-bers**	**Rank (regi-onal project mem-bers)**	**Obser-vers per 1M capita**	**Rank (per 1M capita)**	**Obser-vers per 1,000 km^2^**	**Rank (obser-vers per 1,000 km^2^)**	**Obser-vations per obser-ver**	**Rank (obser-vations per obser-ver)**
Moscow Oblast Flora	1,910	1	88	2	251	9	43.1	4	38.4	41
Flora of Moscow	1,623	2	122	1	129	25	633.7	1	40.8	38
St Petersburg Flora	677	3	20	21	126	26	482.5	2	13.5	71
Leningrad Oblast Flora	642	4	23	17	347	4	7.7	11	15.4	69
Krasnodar Krai Flora	508	5	42	7	90	37	6.7	12	16.8	66
Flora of the Crimea	428	6	59	4	224	12	16.4	5	38.7	39
Nizhny Novgorod Oblast Flora	366	7	21	20	114	31	4.8	19	72.9	16
Sverdlovsk Oblast Flora	345	8	41	10	80	40	1.8	34	53.9	31
Tula Oblast Flora	333	9	64	3	225	11	13.0	6	82.5	14
Irkutsk Oblast Flora	313	10	19	23	131	24	0.4	65	62.8	25
Novosibirsk Oblast Flora	304	11	45	5	109	32	1.7	36	75.4	15
Bashkortostan Flora	302	12	19	22	75	44	2.1	31	53.1	32
Tver Oblast Flora	290	13	25	15	228	10	3.4	24	33.1	46
Tatarstan Flora	276	14	22	19	71	48	4.1	22	64.2	22
Kaluga Oblast Flora	260	15	25	14	258	8	8.7	9	28.5	52
Voronezh Oblast Flora	240	16	16	32	103	33	4.6	20	72.5	17
Vladimir Oblast Flora	234	17	30	12	171	14	8.0	10	62.1	26
Altai Krai Flora	226	18	41	9	97	35	1.3	45	94.2	11
Chelyabinsk Oblast Flora	215	19	24	16	62	55	2.4	30	47.5	35
Flora of Karelia	211	20	11	52	341	5	1.2	47	16.7	67
Sevastopol Flora	199	21	41	8	449	2	230.3	3	110.5	9
Bryansk Oblast Flora	175	22	44	6	146	20	5.0	15	199.5	4
Yaroslavl Oblast Flora	175	23	14	39	139	22	4.8	16	86.2	13
Samara Oblast Flora	170	24	22	18	53	58	3.2	26	50.1	34
Kaliningrad Oblast Flora	163	25	12	47	163	16	10.8	7	47.2	36
Altai Republic Flora	153	26	16	31	699	1	1.6	39	23.3	61
Murmansk Oblast Flora	142	27	15	34	190	13	1.0	52	23.7	60
Krasnoyarsk Krai Flora	140	28	16	30	49	63	0.1	82	60.0	27
Primorsky Krai Flora	138	29	14	38	73	45	0.8	56	56.5	29
Ryazan Oblast Flora	129	30	15	33	116	30	3.3	25	25.7	55
Flora of Khanty-Mansi Autonomous Okrug	126	31	18	25	76	43	0.2	71	63.7	23
Flora of Mordovia	125	32	17	27	157	17	4.8	18	133.2	7
Buryat Republic Flora	121	33	16	29	123	27	0.3	68	30.1	51
Kamchatka Flora	117	34	36	11	372	3	0.3	70	119.4	8
Rostov Oblast Flora	117	35	10	57	28	81	1.2	48	25.0	59
Chuvash Republic Flora	115	36	25	13	94	36	6.3	13	160.9	5
Perm Krai Flora	114	37	16	28	44	67	0.7	57	51.2	33
Stavropol Krai Flora	110	38	8	68	39	72	1.7	38	10.4	79
Volgograd Oblast Flora	107	39	12	46	43	68	0.9	53	32.8	47
Ivanovo Oblast Flora	103	40	10	56	103	34	4.8	17	20.7	63
Kemerovo Oblast Flora	101	41	13	42	38	74	1.1	49	32.7	48
Belgorod Oblast Flora	97	42	18	24	63	54	3.6	23	64.6	21
Pskov Oblast Flora	96	43	9	62	152	19	1.7	35	25.0	58
Novgorod Oblast Flora	87	44	9	61	145	21	1.6	40	21.1	62
Saratov Oblast Flora	87	45	13	41	36	77	0.9	55	38.6	40
Tomsk Oblast Flora	87	46	14	37	81	39	0.3	69	66.2	20
Arkhangelsk Oblast Flora	78	47	8	67	71	47	0.2	73	36.2	43
Flora of Karachay-Cherkessia	77	48	6	74	165	15	5.4	14	34.3	44
Kursk Oblast Flora	77	49	12	45	70	51	2.6	29	342.1	2
Kirov Oblast Flora	75	50	13	40	59	56	0.6	60	70.4	18
Lipetsk Oblast Flora	74	51	4	83	65	52	3.1	28	41.7	37
Flora of Yamalo-Nenets Autonomous Okrug	71	52	17	26	131	23	0.1	77	27.4	54
Flora of Adygea	70	53	10	55	154	18	9.0	8	13.8	70
Tyumen Oblast Flora	69	54	9	60	45	65	0.4	63	101.5	10
Smolensk Oblast Flora	67	55	6	73	71	46	1.3	44	12.8	74
Orenburg Oblast Flora	65	56	8	66	33	79	0.5	62	18.1	65
Penza Oblast Flora	64	57	12	44	49	64	1.5	42	32.3	49
Udmurt Republic Flora	64	58	8	65	42	69	1.5	41	62.9	24
Komi Republic Flora	63	59	3	84	76	42	0.2	75	11.7	76
Ulyanovsk Oblast Flora	63	60	11	51	51	60	1.7	37	38.3	42
Vologda Oblast Flora	58	61	11	49	50	62	0.4	66	33.5	45
Sakhalin Oblast Flora	58	62	11	50	118	29	0.7	59	55.1	30
Kostroma Oblast Flora	57	63	14	36	89	38	0.9	54	223.3	3
Flora of Kabardino-Balkaria	56	64	7	70	65	53	4.5	21	8.7	81
Dagestan Flora	52	65	9	59	17	83	1.0	51	145.9	6
Oryol Oblast Flora	52	66	4	82	70	50	2.1	32	11.0	78
Astrakhan Oblast Flora	51	67	10	54	50	61	1.0	50	9.2	80
Omsk Oblast Flora	51	68	14	35	26	82	0.4	67	387.2	1
Mari El Flora	48	69	7	69	71	49	2.1	33	25.1	57
Khabarovsk Krai Flora	48	70	6	72	36	75	0.1	81	12.7	75
Tambov Oblast Flora	42	71	5	78	41	71	1.2	46	25.5	56
Flora of Khakassia	41	72	11	48	76	41	0.7	58	27.7	53
Flora of Yakutia	41	73	10	53	42	70	0.0	85	12.8	73
Zabaykalsky Krai Flora	33	74	8	64	31	80	0.1	79	31.0	50
Amur Oblast Flora	30	75	9	58	38	73	0.1	78	86.7	12
Kurgan Oblast Flora	29	76	12	43	35	78	0.4	64	67.9	19
Flora of North Ossetia	25	77	5	77	36	76	3.1	27	5.1	84
Tyva Republic Flora	18	78	6	71	55	57	0.1	76	11.4	77
Magadan Oblast Flora	17	79	5	76	120	28	0.0	83	13.0	72
Flora of Chukotka	16	80	8	63	322	6	0.0	84	58.0	28
Flora of Kalmykia	14	81	2	85	51	59	0.2	74	3.4	85
Flora of Nenets Autonomous Okrug	12	82	4	81	274	7	0.1	80	6.8	82
Chechen Republic Flora	9	83	4	80	6	85	0.6	61	19.4	64
Flora of Jewish Autonomous Oblast	7	84	4	79	44	66	0.2	72	15.7	68
Flora of Ingushetia	5	85	5	75	10	84	1.4	43	6.4	83

**Table 16.** Top observers of the regional projects (a - author, c - contributor)

**Regional project**	**Top observer (observations)**	**Number of observa- tions**	**Top observer (species)**	**Number of species**
Flora of Moscow	A.P. Seregin (apseregin), a	14,900	A.P. Seregin (apseregin), a	791
Bryansk Oblast Flora	N.N. Panasenko (panasenkonn), c	13,348	N.N. Panasenko (panasenkonn), c	1,010
Kursk Oblast Flora	N.I. Degtyarev (dni_catipo), a	10,966	N.I. Degtyarev (dni_catipo), a	841
Chuvash Republic Flora	S.M. Appolonov (velibortravoved), c	10,676	S.M. Appolonov (velibortravoved), c	577
Omsk Oblast Flora	V.I. Teplouhov (vladimir_teplouhov), a	9,894	V.I. Teplouhov (vladimir_teplouhov), a	492
Sevastopol Flora	E.S. Kashirina (katerina_kashirina), a	8,830	S.A. Svirin (sapsan), a	1,050
Yaroslavl Oblast Flora	E.V. Garin (eduard_garin), a	6,351	E.V. Garin (eduard_garin), a	669
Altai Krai Flora	P.V. Golyakov (pavel_golyakov), a	6,285	P.V. Golyakov (pavel_golyakov), a	829
Moscow Oblast Flora	N.V. Ivanova (dryomys)	6,235	V.Y. Arkhipov (vladimirarkhipov), a	561
Krasnoyarsk Krai Flora	I.N. Pospelov (taimyr), a	5,608	I.N. Pospelov (taimyr), a	659
Kaliningrad Oblast Flora	N.P. Zelenova (npz), a	5,539	N.P. Zelenova (npz), a	543
Kamchatka Flora	O.P. Kuryakova (olga2019kuryakova), a	5,154	B.V. Bolshakov (borisbolshakov), a	553
Tatarstan Flora	V.E. Prokhorov (vadim_prokhorov), a	5,001	V.E. Prokhorov (vadim_prokhorov), a	822
Nizhny Novgorod Oblast Flora	T.V. Zarubo (tatyanazarubo), a	4,573	T.V. Zarubo (tatyanazarubo), a	591
Kostroma Oblast Flora	S.A. Nesterova (ledum), a	4,385	S.A. Nesterova (ledum), a	719
Novosibirsk Oblast Flora	A.P. Zyrianov (alzov), a	4,268	K.V. Romanov (kildor), a	566
Irkutsk Oblast Flora	S.V. Mirvoda (smsergey), a	4,222	A.V. Verkhozina (allaverkhozina), a	627
Chelyabinsk Oblast Flora	Y.O. Magazov (yaroslavmagazov), a	4,027	Y.O. Magazov (yaroslavmagazov), a	387
Voronezh Oblast Flora	A.N. Khimin (aleks-khimin), a	3,782	A.N. Khimin (aleks-khimin), a	626
Tomsk Oblast Flora	A.L. Ebel (aleksandrebel), a	3,579	A.L. Ebel (aleksandrebel), a	643
Dagestan Flora	M.M. Mallaliev (mallaliev), a	3,508	R.A. Murtazaliev (ramazan_murtazaliev), a	1,594
Samara Oblast Flora	D.V. Tretyakova (divitre), a	3,424	D.V. Tretyakova (divitre), a	469
Tyumen Oblast Flora	Y.M. Basov (yurii_basov), a	3,384	Y.M. Basov (yurii_basov), a	469
Flora of Mordovia	A.A. Khapugin (hapugin88), a	3,322	A.A. Khapugin (hapugin88), a	536
Sverdlovsk Oblast Flora	D.V. Nesterkova (dinanesterkova), a	3,151	M.S. Knjazev (mihail13), a	661
Primorsky Krai Flora	V.S. Volkotrub (vvolkotrub), c	3,052	V.S. Volkotrub (vvolkotrub), c	1,238
Bashkortostan Flora	E. Ishmukhametova (evelina_ishmukhametova)	2,952	E. Ishmukhametova (evelina_ishmukhametova)	467
Belgorod Oblast Flora	V.N. Zelenkova (sesquicentennial), a	2,784	V.N. Zelenkova (sesquicentennial), a	645
Flora of the Crimea	E.A. Razina (lenatara)	2,648	E.A. Razina (lenatara)	983
Flora of Khanty-Mansi Autonomous Okrug	N.V. Filippova (ninacourlee), a	2,579	N.V. Filippova (ninacourlee), a	352
Vladimir Oblast Flora	V.V. Stepanov (vist), a	2,357	A.P. Seregin (apseregin), a	609
Sakhalin Oblast Flora	S.A. Nesterova (ledum), a	2,094	S.A. Nesterova (ledum), a	574
Saratov Oblast Flora	A.N. Kandaurova (cava), a	1,964	A.N. Kandaurova (cava), a	483
Perm Krai Flora	M.E. Trubinova (mashat), c	1,843	I.V. Pavlov (pavloviv), a	337
Leningrad Oblast Flora	M.I. Ismaylov (maxim_ismaylov), c	1,667	A. Kondratieva (alina_kondratieva)	407
Kirov Oblast Flora	V. Bryukhov (woodmen19), c	1,624	V. Bryukhov (woodmen19), c	450
St Petersburg Flora	M.I. Ismaylov (maxim_ismaylov), c	1,552	A. Kondratieva (alina_kondratieva)	359
Buryat Republic Flora	D.G. Chimitov (daba), a	1,547	D.G. Chimitov (daba), a	506
Kaluga Oblast Flora	A.A. Malyutkin (sansan_94)	1,429	N.V. Ivanova (dryomys)	335
Flora of Karachay-Cherkessia	D.A. Bochkov (convallaria1128), a	1,362	D.A. Bochkov (convallaria1128), a	599
Arkhangelsk Oblast Flora	G. Okatov (gen_ok)	1,314	G. Okatov (gen_ok)	660
Rostov Oblast Flora	S.R. Mayorov (phlomis_2019), a	1,301	S.R. Mayorov (phlomis_2019), a	510
Tula Oblast Flora	T.Y. Svetasheva (tsvetasheva), a	1,294	T.Y. Svetasheva (tsvetasheva), a	529
Tver Oblast Flora	E.S. Pushay (pushai), a	1,108	A.P. Seregin (apseregin), a	370
Amur Oblast Flora	A.P. Seregin (apseregin), a	1,052	S.A. Nesterova (ledum), a	367
Udmurt Republic Flora	S. Seleznev (sergejseleznev)	1,006	S. Seleznev (sergejseleznev)	367
Kemerovo Oblast Flora	E.E. Perfilev (gyng), c	970	E.E. Perfilev (gyng), c	410
Ryazan Oblast Flora	P.Y. Likhacheva (polinalikhacheva), c	948	P.Y. Likhacheva (polinalikhacheva), c	267
Kurgan Oblast Flora	Y.M. Basov (yurii_basov), a	936	Y.M. Basov (yurii_basov), a	250
Ulyanovsk Oblast Flora	A.V. Korobkov (korobkov)	777	R. Anashkina (rimma_anashkina)	333
Vologda Oblast Flora	D.A. Filippov (dmitriy_philippov), c	754	D.A. Filippov (dmitriy_philippov), c	279
Krasnodar Krai Flora	A.P. Seregin (apseregin), a	712	Y.V. Danilevsky (yuriydanilevsky), a	339
Pskov Oblast Flora	E.S. Popov (epopov), a	641	E.S. Popov (epopov), a	386
Flora of Karelia	anonymous (plrays)	626	anonymous (plrays)	234
Lipetsk Oblast Flora	S.Y. Korovaicev (mrsalento), c	617	A.P. Seregin (apseregin), a	355
Murmansk Oblast Flora	D.A. Bochkov (convallaria1128), a	593	D.A. Bochkov (convallaria1128), a	242
Penza Oblast Flora	D. Polikanin (zemleved)	576	D. Polikanin (zemleved)	345
Volgograd Oblast Flora	A.P. Seregin (apseregin), a	555	A.P. Seregin (apseregin), a	334
Mari El Flora	V.A. Bakutov (vladimirbakutov), a	553	V.A. Bakutov (vladimirbakutov), a	359
Tambov Oblast Flora	E. Yarova (hln_m_t)	524	E. Yarova (hln_m_t)	196
Flora of Chukotka	I.N. Pospelov (taimyr), a	469	I.N. Pospelov (taimyr), a	159
Altai Republic Flora	N.V. Filippova (ninacourlee), a	430	K.V. Romanov (kildor), a	170
Flora of Khakassia	A.L. Ebel (aleksandrebel), a	353	A.L. Ebel (aleksandrebel), a	209
Ivanovo Oblast Flora	anonymous (olia27)	309	E. Voinova (ekaterinavoinova)	205
Novgorod Oblast Flora	N. Zouieva (nat_zouieva)	302	A. Nikanorova (feanaro)	166
Zabaykalsky Krai Flora	D.G. Chimitov (daba), a	281	D.G. Chimitov (daba), a	165
Flora of Yamalo-Nenets Autonomous Okrug	I.N. Pospelov (taimyr), a	252	I.N. Pospelov (taimyr), a	136
Stavropol Krai Flora	M.A. Orlov (naturalist16000), c	237	M.A. Orlov (naturalist16000), c	140
Astrakhan Oblast Flora	A. Golovchenko (alena_golovchenko)	224	A. Golovchenko (alena_golovchenko)	93
Flora of Adygea	E. Shaw (ed_shaw)	163	E. Shaw (ed_shaw)	110
Komi Republic Flora	E. Shubnitsina (elena_sh)	156	E. Shubnitsina (elena_sh)	89
Flora of Yakutia	C. Rixen (christianrixen)	134	C. Rixen (christianrixen)	106
Flora of Kabardino-Balkaria	M.P. Shashkov (max_carabus)	134	M.P. Shashkov (max_carabus)	70
Orenburg Oblast Flora	V.P. Travkin (vladimirtravkin), a	131	V.P. Travkin (vladimirtravkin), a	87
Smolensk Oblast Flora	D. Kulakova (daria_kulakova)	124	D. Kulakova (daria_kulakova)	112
Oryol Oblast Flora	M. Frolenkova (frolenkovamar)	108	M. Frolenkova (frolenkovamar)	66
Tyva Republic Flora	A.I. Pyak (pyakai), a	100	A.I. Pyak (pyakai), a	95
Khabarovsk Krai Flora	A. Sukhinina (sukhinina-a)	86	V.A. Belova (veronika_belova), a	65
Flora of Jewish Autonomous Oblast	V.A. Belova (veronika_belova), a	62	V.A. Belova (veronika_belova), a	51
Chechen Republic Flora	T.A. Avtaeva (tomaavtaeva)	60	T.A. Avtaeva (tomaavtaeva)	56
Magadan Oblast Flora	I.N. Pospelov (taimyr), a	42	E. Yusupova (ekaterina_yusupova)	36
Flora of Nenets Autonomous Okrug	D.G. Ivanov (ivanovdg19), c	32	D.G. Ivanov (ivanovdg19), c	25
Flora of North Ossetia	V.N. Korotkov (vladimir_korotkov), a	23	V.N. Korotkov (vladimir_korotkov), a	22
Flora of Ingushetia	R.A. Murtazaliev (ramazan_murtazaliev), a	19	R.A. Murtazaliev (ramazan_murtazaliev), a	19
Flora of Kalmykia	V.E. Prokhorov (vadim_prokhorov), a	12	V.E. Prokhorov (vadim_prokhorov), a	11
